# Celastrol in Rheumatoid Arthritis: Immunomodulatory Mechanisms, Multicellular Convergence, and Translational Challenges

**DOI:** 10.3390/ijms27198834

**Published:** 2026-10-03

**Authors:** Fernanda-Isadora Corona-Meraz, Beatriz Teresita Martín-Márquez, Luis E. Cobos-Puc, Elisa Guzmán-Alemán, Hilda Aguayo-Morales, Luis Daniel Hernández-Ortega, Roberto Carlos Rosales-Gómez, Rolando Castañeda-Arellano, Flavio Sandoval-García, Alvaro Jovanny Tovar-Cuevas

**Affiliations:** 1 Universidad de Guadalajara, Centro Universitario de Tonalá, Departamento de Ciencias Biomédicas, Centro de Investigación Multidisciplinario en Salud, Tonalá C.P. 45425, Jalisco, Mexico; mariaf.corona@academicos.udg.mx (F.-I.C.-M.); luis.hortega@academicos.udg.mx (L.D.H.-O.); roberto.rosales@academicos.udg.mx (R.C.R.-G.); rolando.castaneda@academicos.udg.mx (R.C.-A.); 2 Universidad de Guadalajara, Centro Universitario de Ciencias de la Salud, Departamento de Biología Molecular y Genómica, Instituto de Investigación en Reumatología y Sistema Músculo Esquelético, Guadalajara C.P. 44340, Jalisco, Mexico; beatriz.martin@academicos.udg.mx (B.T.M.-M.); flavio.sandoval@academicos.udg.mx (F.S.-G.); 3 Universidad de Guadalajara, Centro Universitario de Ciencias de la Salud, Departamento de Biología Molecular y Genómica, Cuerpo Académico UDG-CA-703 “Inmunología y Reumatología”, Guadalajara C.P. 44340, Jalisco, Mexico; 4 Facultad de Ciencias Químicas, Universidad Autónoma de Coahuila, Boulevard V. Carranza S/N, Saltillo C.P. 25280, Coahuila, Mexico; luis.cobos@uadec.edu.mx (L.E.C.-P.); aguayohilda@uadec.edu.mx (H.A.-M.); 5 Laboratorio de Análisis Clínicos, El Hospital Regional de Alta Especialidad de Tlajomulco de Zúñiga del ISSSTE, Tlajomulco C.P. 45640, Jalisco, Mexico; guzalely@gmail.com

**Keywords:** celastrol, rheumatoid arthritis, immunomodulation, fibroblast-like synoviocytes, osteoclastogenesis, cellular crosstalk

## Abstract

Rheumatoid arthritis (RA) arises from interactions among immune, stromal, and osteoimmune compartments, supporting interest in agents with multilevel activity, such as celastrol. This narrative review critically integrates evidence on its chemical–pharmacological basis, immunomodulatory mechanisms, cellular effects, and translational potential in RA. Preclinical data show inhibition of inflammatory programs in macrophages and neutrophils, modulation of T- and B-cell responses, suppression of pathogenic fibroblast-like synoviocyte (FLS) functions, and reduced osteoclastogenesis and structural damage. COMM domain-containing proteins 3 and 8 (COMMD3/8) provide the strongest causal validation, while insulin-like growth factor 2 mRNA-binding protein 3 (IGF2BP3) has biophysical and functional support; numerous inflammatory and metabolic nodes primarily represent downstream signaling. Multicellular models support functional convergence, but intercellular causation in RA remains limited; directional functional evidence for dendritic cell–T-cell modulation comes from non-rheumatoid models, and no persistent synovial reprogramming has been demonstrated. Low bioavailability, multitarget covalent pharmacology, a narrow therapeutic window, and contributions from delivery carriers constrain translational interpretation. Addressing these translational challenges explicitly can help guide future research to improve celastrol’s safety and efficacy, which is essential for clinical application.

## 1. Introduction

Rheumatoid arthritis (RA) is an inflammatory, immune-mediated, and destructive disease whose persistence depends on interactions among interdependent cellular compartments. Within the inflamed synovium, multiple cellular populations contribute to disease persistence and progression through distinct yet convergent mechanisms. Synovial monocytes and macrophages sustain production of inflammatory mediators, whereas neutrophils contribute reactive oxygen species (ROS) and structures associated with neutrophil extracellular traps (NETs) [1]. Antigen-presenting cells, together with T and B lymphocytes, promote adaptive immune responses. At the same time, fibroblast-like synoviocytes (FLSs) acquire proliferative, migratory, and invasive properties that further contribute to synovial inflammation and tissue remodeling [2,3]. Osteoclasts, in turn, integrate immune- and stroma-derived signals to mediate bone erosion. Together, these multicellular contributions illustrate the complexity of rheumatoid arthritis pathogenesis and show that disease activity and joint destruction cannot be attributed to a single cell type or isolated mediator [4].

The convergence of multiple cell types onto shared intracellular pathways, such as NF-κB, MAPKs, mTOR, or redox signaling, drives a shared inflammatory phenotype; however, this shared activation does not inherently demonstrate causal intercellular crosstalk [5]. Similarly, a simultaneous reduction in inflammation, infiltration, and erosion does not allow the identification of the compartment that initiates the pharmacological effect. Accordingly, this review distinguishes parallel cellular effects, mechanistic convergence, and experimentally supported disruption of pathogenic intercellular circuits.

Celastrol, also known as tripterine, is a pentacyclic quinone methide triterpenoid isolated from *Tripterygium wilfordii* Hook F. and is distinct from whole extracts and multicomponent botanical preparations [6,7]. Celastrol’s quinone methide moiety acts as an electrophilic Michael acceptor that can modify nucleophilic cysteine residues. This reactivity may contribute to its biological activity but also makes it difficult to distinguish selective target engagement from broader, less selective modification of cellular proteins.

Translational development is further constrained by poor aqueous solubility, limited and variable oral exposure, nonselective distribution, and a relatively narrow therapeutic window [8,9,10].

Preclinical studies report celastrol-associated effects across innate and adaptive immune cells, fibroblast-like synoviocytes, and osteoclast-related responses, although the depth of mechanistic support varies substantially among compartments. Some findings integrate physical interaction or target engagement with functional validation, whereas others remain limited to downstream pathway changes, phenotypic associations, or formulation-dependent effects. Multicellular models can reproduce selected features of synovial organization and cell–cell interactions [11,12], but they do not necessarily establish the direction, necessity, or persistence of the intercellular signals involved. Accordingly, free celastrol, formulation-based effects, target engagement, functional inhibition, and nonspecific loss of viability require separate interpretation.

The central question is whether the reported preclinical effects of celastrol reflect convergent, multilevel modulation of the synovial microenvironment or whether that interpretation remains largely based on indirect, formulation-dependent, and predominantly preclinical evidence.

## 2. Search Strategy and Review Approach

We conducted a structured narrative review, searching PubMed/MEDLINE, Embase, Scopus, Web of Science Core Collection, and ClinicalTrials.gov, focusing on the literature published from January 2020 onward, with selective inclusion of earlier seminal studies and relevant recent publications identified during manuscript preparation. The searches were last updated on 6 September 2026. Search concepts included celastrol, rheumatoid arthritis, immune populations, fibroblast-like synoviocytes, osteoclasts, crosstalk, signaling, pharmacokinetics, toxicity, and delivery systems.

Original studies using purified celastrol, human evidence, and models directly related to RA were prioritized. Preclinical models were used to interpret mechanisms and assess pharmacological plausibility; evidence for other diseases was considered extrapolative. Multicomponent extracts without specific attribution to celastrol, related compounds, theses, preprints, unpublished manuscripts, and the gray literature were excluded. Evidence was qualitatively evaluated based on disease relevance, the depth and directness of mechanistic support, and the pharmacological plausibility of the experimental exposure. 

The synthesis distinguished formulation-free celastrol and separated physical binding, target engagement, functional validation, downstream changes, and computational predictions. Pre-2020 references were retained when they provided mechanistic underpinnings or core experimental evidence.

Two coauthors independently developed methodological proposals, which were then compared and integrated into a shared framework. This framework established criteria for selecting studies, assessing experimental relevance to RA, classifying mechanistic evidence, and interpreting pharmacological findings. Mechanistic directness was distinguished from disease relevance, and multicellular activity was evaluated separately from experimentally demonstrated intercellular causation. The framework also differentiated the effects of free celastrol from those potentially attributable to delivery systems. Under this framework, literature searches, study selection, and qualitative evidence assessment were conducted collaboratively, with search concepts iteratively refined as new mechanistic and translational questions emerged. For the qualitative synthesis, study-level information was organized by experimental system, celastrol intervention and formulation, mechanistic methods, relevant controls, principal outcomes, and key limitations, as applicable.

## 3. Chemical–Pharmacological Bases and Translational Barriers of Celastrol

Celastrol is a pentacyclic quinone methide triterpenoid with the molecular formula C_29_H_38_O_4_ and a molecular mass of 450.6 g/mol. Its hydrophobic triterpenoid scaffold features a conjugated quinone methide system in ring A, a carboxyl group at C-20, and a hydroxyl group at C-6. Experimental studies report very low aqueous solubility, approximately 0.044 mg/mL, and a high oil–water partition coefficient (log P = 5.63), consistent with substantial lipophilicity and formulation constraints [13,14]. The quinone methide moiety serves as an electrophilic Michael acceptor capable of reacting with nucleophilic cysteine residues. Comparative structure–activity studies indicate that modifying this reactive moiety can markedly reduce biological activity in specific experimental systems, supporting a role for electrophilic reactivity in celastrol pharmacology without establishing a universal structure–activity relationship [15]. Chemical modification at C-6 and at the C-20 carboxyl group has also produced substantial changes in biological activity, indicating that peripheral substitution can modify the pharmacological profile of the celastrol scaffold [15,16]. Covalent reactivity therefore contributes to celastrol’s pharmacological profile but, by itself, does not establish molecular selectivity.

Among the proposed molecular targets of celastrol, COMM domain-containing proteins 3 and 8 (COMMD3/8) currently have the strongest mechanistic support. Identification of a covalently modified cysteine residue, together with loss of celastrol responsiveness in a resistant variant, links physical interaction to cellular and functional causality [17]. IGF2BP3 is supported by surface plasmon resonance, colocalization of biotinylated celastrol, and pull-down assays using RA-FLS, RAW264.7 cell lysates, and RA synovial tissue lysates. Functional and genetic evidence further links IGF2BP3 to the RASGRF1–mTORC1 axis and to celastrol-mediated anti-arthritic activity. However, definitive target engagement by unmodified celastrol in intact human RA cells remains to be established [18]. Histone deacetylase 3 (HDAC3) is supported by cell-associated thermal stabilization, lysate-based microscale thermophoresis, and functional and mutational evidence linking its modulation to the forkhead box O3 (FOXO3)–glutaminase 1 (GLS1) axis and to changes in glutaminolysis and T helper 17 (Th17) cell differentiation. However, this evidence derives from murine T-cell and colitis systems rather than from RA-specific models or cells from patients with RA [19]. For the inhibitor of nuclear factor kappa B kinase beta (IKKβ), dose-dependent biochemical inhibition, resistance of the C179A mutant, and reversal of inhibition by dithiothreitol provide substantial biochemical and mutational evidence that Cys179 is critical for celastrol sensitivity and support a cysteine-dependent interaction. However, researchers did not report the direct identification of a celastrol–IKKβ adduct or cellular target-engagement measurements [20]. For NADPH oxidase 2 (NOX2), direct binding of celastrol to recombinant p47phox was supported by intrinsic fluorescence and native mass spectrometry. Disruption of the p47phox–p22phox interaction and inhibition of NOX2-dependent ROS production provided biochemical and cellular functional support. However, the binding site and chemical nature of the celastrol–p47phox interaction, cellular target engagement, and dependence of anti-arthritic efficacy on p47phox or NOX2 were not established [21]. Network pharmacology, transcriptomic analyses, and molecular docking identified arachidonate 5-lipoxygenase (ALOX5) as a candidate target, whereas single-cell analyses revealed enrichment of ALOX5 expression in macrophages [22]. In contrast, changes in the phosphorylation, translocation, expression, or activity of NF-κB, MAPK, NLR family pyrin domain-containing 3 (NLRP3), signal transducer and activator of transcription 3 (STAT3), mTOR, nuclear factor of activated T cells 1 (NFATc1), and related pathways primarily document downstream functional modulation and do not establish direct binding.

For this review, we organized mechanistic evidence into a review-derived qualitative hierarchy encompassing physical binding, target engagement in the relevant cell, functional validation, downstream association, and computational prediction, informed by established approaches to target identification and cellular target engagement [23,24,25]. Table 1 summarizes the relative positions of the principal candidate targets and mechanisms. These levels reflect the depth of mechanistic evidence and are not intended as a quantitative score or measure of clinical relevance. Evidence categories in Table 1 were assigned based on the experimental approaches reported in the original studies. Physical interaction was supported by direct biochemical or biophysical binding assays, whereas cellular target engagement required evidence of interaction with the proposed target in a cellular context. Functional effects on target-associated processes were distinguished from causal validation, which required target-specific perturbation, resistance, or rescue experiments linking the proposed target to celastrol responsiveness. Changes in protein expression or signaling without demonstrated binding were classified as downstream modulation, whereas computational predictions alone were considered hypothesis-generating. Multiple categories were retained when experimentally supported, and relevance to RA was assessed independently of mechanistic directness.

Overall, celastrol is better understood as a context-dependent electrophilic modifier with demonstrated covalent reactivity than as a conventional selective ligand. The term multitarget should therefore be used cautiously, as the available evidence comprises a limited number of comparatively well-supported molecular interactions alongside broader downstream changes in inflammatory, stress-response, survival, and metabolic pathways. In rheumatoid arthritis fibroblast-like synoviocytes (RA-FLSs), functional effects observed at submicromolar-to-micromolar concentrations, along with greater cell death at higher exposures, suggest a concentration-dependent shift from pathway modulation toward cellular stress and cytotoxicity [27,41]. Because experimental systems differ substantially, no universal concentration threshold exists. Other stress-response pathways remain model-dependent and should not be weighted equally with validated molecular interactions or with functional genetic and rescue evidence.

The pharmacokinetics of free celastrol further constrain the translational interpretation of in vitro findings. Oral exposure is limited and varies substantially across experimental conditions, with absolute oral bioavailability reported at about 3% and 17% in rat studies under different dosing and formulation conditions [9,10]. Reported mean elimination half-lives after oral administration of pure celastrol were 12.02 ± 8.32 h (n = 3) [9] and 10.20 ± 2.17 h in female rats (n = 6) [10]. However, differences in dose, formulation vehicle, sampling, and experimental design limit their generalizability. Celastrol also undergoes extensive biotransformation through multiple oxidative and conjugative pathways, involving both hepatic and intestinal metabolism [42]. Differences in dose, route, formulation, sampling schedule, and analytical methods limit direct comparisons of maximum plasma concentration (Cmax), area under the concentration–time curve (AUC), clearance, and volume of distribution across studies. Available data therefore support limited and variable systemic exposure after oral administration, but do not permit a single quantitative exposure estimate that generalizes across experimental settings. Selective joint exposure to free celastrol remains insufficiently characterized. A 2026 collagen-induced arthritis (CIA) study comparing an oral self-microemulsifying celastrol formulation with a celastrol suspension at the same nominal dose reported greater anti-arthritic efficacy with the formulation [43]. However, plasma and joint celastrol concentrations were not measured, so these findings demonstrate formulation-dependent efficacy rather than improved oral bioavailability or increased systemic or synovial celastrol exposure.

Across preclinical arthritis models, celastrol has been associated with reduced inflammatory activity and structural damage, although treatment timing, exposure, and experimental design vary considerably [28,44,45]. Preventive studies demonstrate interference with disease development, whereas therapeutic regimens more directly address established inflammation. Reduced erosion and preservation of joint structure support a protective effect, but they do not, by themselves, demonstrate repair of pre-existing damage or durable disease modification [44,45]. Translational interpretation is further constrained by the relatively narrow therapeutic window reported for free celastrol. Systemic toxicity has been reported at higher exposures, and in adjuvant-induced arthritis (AIA), effective oral doses approached those associated with thymic or hepatic alterations [8,44]. Searches for this review did not identify any ClinicalTrials.gov registration evaluating celastrol as an intervention for patients with RA. Evidence from multicomponent T. wilfordii preparations, Kunxian, triptolide, pristimerin, and celastrol derivatives may provide relevant pharmacological context but do not constitute direct evidence for purified celastrol. The relationships among chemical reactivity, the hierarchy of molecular evidence, exposure, delivery systems, and translational barriers are summarized in Figure 1.

## 4. Innate Immunity

### 4.1. Monocytes and Macrophages

Monocytes and macrophages link synovial inflammation to structural damage because infiltrating monocytes can differentiate into macrophages or osteoclasts in response to macrophage colony-stimulating factor (M-CSF) and receptor activator of nuclear factor κB ligand (RANKL). The abundance of cluster of differentiation 68-positive (CD68^+^) macrophages in the synovial sublining is associated with inflammatory disease activity. In AIA, celastrol reduced synovial cellularity and the abundance of CD68^+^ macrophages, preserved joint architecture, and limited bone damage [45], whereas in collagen-induced arthritis (CIA), distinct celastrol formulations reduced synovitis, erosion, and cartilage degradation [28,32].

Among the signaling pathways evaluated in macrophages, NF-κB is one of the most consistently observed functional nodes. In LPS-stimulated RAW264.7 macrophages, free celastrol and celastrol-loaded formulations reduced phosphorylation of the NF-κB p65 subunit (p65) and IκBα, as well as p65 nuclear translocation, and decreased iNOS and pro-inflammatory cytokines. IL-10 and arginase 1 were increased under the same experimental conditions, with the targeted formulation generally producing greater effects than free celastrol [32]. In monocyte-derived human macrophages, celastrol decreased NF-κB, p38 mitogen-activated protein kinase (p38), cyclooxygenase-2, microsomal prostaglandin E synthase-1, and pro-inflammatory lipid mediators [47]. These measurements are consistent with functional inhibition of inflammatory pathways, although the underlying molecular interaction remains undefined.

A second layer of functional evidence in macrophage-related models involves the modulation of inflammasome-associated responses. In macrophages derived from THP-1 and U937 cells and stimulated with the parvovirus B19 nonstructural protein 1 (NS1), celastrol decreased protein levels of NLRP3, apoptosis-associated speck-like protein containing a caspase recruitment domain (ASC), caspase-1, and IL-18 and reduced IL-1β release [35]. These findings are consistent with the suppression of NLRP3-associated inflammasome signaling, but they provide insufficient evidence of direct NLRP3 inhibition and have not been validated in primary synovial macrophages from patients with RA. Moreover, the viral NS1 stimulus does not replicate the autoimmune synovial microenvironment.

Studies using celastrol-containing systems have reported changes in macrophage-associated phenotypic markers. In lipopolysaccharide (LPS)-stimulated RAW264.7 cells, celastrol-containing treatments decreased cluster of differentiation 86 (CD86) and inducible nitric oxide synthase (iNOS) and increased cluster of differentiation 206, arginase-1, and IL-10. The targeted formulation generally produced stronger effects than free celastrol [32]. An inflammation-targeted celastrol nanodrug likewise reduced features associated with the M1-like macrophage phenotype (M1) in RA-related experimental models [28]. These findings align with shifts in markers associated with M1-like and M2-like macrophage phenotypes (M1 and M2, respectively), rather than demonstrating stable macrophage repolarization. Importantly, these experiments primarily assessed marker profiles during phenotype induction and did not establish persistent functional conversion of previously established M1 macrophages. In human monocyte-derived macrophages undergoing IL-4-induced polarization, celastrol reduced 12/15-lipoxygenase products and specialized pro-resolving mediators, indicating that changes toward M2-associated markers do not necessarily imply acquisition of a complete resolving phenotype [47]. At a viability-preserving concentration, a self-microemulsifying celastrol formulation reduced tumor necrosis factor alpha (TNF-α) and IL-1β expression more than free celastrol and increased IL-10 in LPS-stimulated RAW264.7 macrophages [43]. However, at higher concentrations, the formulation was more cytotoxic than free celastrol, supporting formulation-dependent anti-inflammatory activity without establishing stable macrophage repolarization.

As a complementary therapeutic approach, targeted celastrol delivery systems have been used to promote apoptosis in pathogenic cell populations. An MMP9-responsive celastrol nanoparticle system targeting inflammatory macrophages and osteoclasts increased apoptosis in both populations and was associated with reduced inflammatory activity and attenuated bone erosion in experimental arthritis [48]. Similarly, synovial macrophage-targeted celastrol nanoemulsions promoted macrophage apoptosis, shifted the balance between apoptosis and proliferation, and reduced inflammatory and structural disease manifestations [49]. These studies provide evidence for preferential targeting at the formulation level, but they do not establish that celastrol itself selectively induces apoptosis in pathogenic macrophages or osteoclasts. Accordingly, the observed effects should be attributed to the compound–carrier systems, and the quantitative separation between therapeutically intended apoptosis and nonspecific celastrol-associated cytotoxicity remains incompletely defined.

Additional mechanistic insight comes from the IGF2BP3/RASGRF1–mTORC1 axis. Biophysical interaction with IGF2BP3, functional perturbation in RA-FLS and macrophage models, and the absence of additional celastrol-mediated protection in IGF2BP3-deficient arthritic mice provide convergent biophysical, functional, and genetic support for this mechanism [18]. ALOX5 is a substantially less validated candidate mechanism. Transcriptomic, network-pharmacology, and single-cell analyses identified ALOX5 as an RA-associated node enriched in macrophages and linked its expression to TNF–NF-κB signaling and matrix metalloproteinase-associated responses; molecular docking further predicted a potential celastrol–ALOX5 interaction. However, researchers have not demonstrated direct celastrol–ALOX5 binding, cellular target engagement, or experimental validation of a macrophage-specific, ALOX5-dependent therapeutic mechanism [22].

Targeted delivery systems may enhance the apparent therapeutic performance of celastrol while complicating attribution of the observed effects. ROS- or matrix metalloproteinase-2 (MMP-2)-responsive micelles, neutrophil membrane coatings, and scavenger receptor-targeting ligands have been associated with increased macrophage uptake and reduced apparent toxicity. More recently, folate-modified celastrol liposomes were compared with free celastrol and non-targeted celastrol liposomes in macrophage and CIA models. Folate competition reduced cellular uptake, supporting receptor-mediated targeting, while the targeted formulation showed greater apparent anti-inflammatory activity and joint accumulation than the comparator preparations [50]. However, biodistribution was assessed qualitatively, and celastrol pharmacokinetics were not measured. These findings support formulation-dependent delivery rather than demonstrating greater systemic or synovial exposure to free celastrol. More broadly, some carriers can independently scavenge ROS, modulate nitric oxide, or influence macrophage polarization. Appropriate empty-carrier controls, free-celastrol comparators, non-targeted formulations, and exposure-matched joint measurements therefore remain important for distinguishing celastrol pharmacology from delivery-system effects [28,32,50,51].

Important uncertainties remain regarding recruitment, efferocytosis, persistence of the observed phenotype, and selectivity for pathogenic myeloid populations [45,48]. Reduced cell viability may itself decrease inflammatory mediators, while the simultaneous reductions in both pro-inflammatory and pro-resolving mediators in human macrophages indicate that a simple M1/M2 framework is insufficient to capture the observed responses [47,52]. Overall, the available evidence supports attenuation of myeloid inflammatory programs and improvement in experimental arthritis [30,45]. In contrast, causal inflammasome inhibition, selective apoptosis of pathogenic populations, and stable acquisition of a pro-resolving phenotype remain less firmly established. Accordingly, reductions in p65 activation, cytokine production, or CD68^+^ macrophage abundance are consistent with attenuated inflammatory activity but are insufficient to demonstrate persistent macrophage reprogramming [45,47].

### 4.2. Neutrophils: ROS and Structures Associated with NETs

Neutrophils contribute to RA pathogenesis by producing ROS, forming NETs, and releasing inflammatory mediators, which can promote synovial damage and increase exposure to citrullinated autoantigens. However, we found no eligible studies of celastrol in neutrophils isolated directly from patients with RA. The closest human evidence derives from neutrophils obtained from healthy donors and stimulated with immunoglobulin G (IgG) immune complexes or TNF-α, experimental conditions that reproduce selected RA-relevant stimuli but not the persistent phenotype and complex microenvironment of synovial neutrophils in RA [33,53]. In this RA-relevant neutrophil model, free celastrol inhibited the oxidative burst and reduced the release of NET-associated structures. The reduction in ROS was accompanied by decreased phosphorylation of spleen tyrosine kinase (SYK), mitogen-activated protein kinase kinase (MEK), extracellular signal-regulated kinase (ERK), and IκBα, as well as reduced histone H3 citrullination [33]. These results support an effect on the SYK–MEK–ERK–NF-κB cascade but do not identify SYK as a direct target. The most specific biochemical evidence comes from a cell-free system and from healthy human neutrophils, where celastrol reduced NADPH oxidase 2 (NOX2) activity and altered the interaction between the 47 kDa phagocyte oxidase subunit (p47phox) and the 22 kDa phagocyte oxidase subunit (p22phox). 

Direct binding of celastrol to recombinant p47phox was supported by intrinsic fluorescence and native mass spectrometry, and celastrol disrupted the p47phox–p22phox interaction in a pull-down assay. However, the binding site and the chemical nature of the interaction were not established, and the authors did not demonstrate cellular target engagement, mutational validation, or functional rescue in RA-relevant cells [21]. The inhibitory effect of celastrol on NET-associated responses was reproduced across several stimuli. In one study, activity was observed at 0.3125–1.25 µM without detectable cytotoxicity, whereas another reported largely preserved viability up to 10 µM. The half-maximal inhibitory concentrations for the oxidative burst were 0.34 µM in response to TNF-α and 1.53 µM in response to immune complexes. In comparison, phosphorylation and histone citrullination were also assessed at 5–10 µM. Cytotoxicity was detected at 5 µM in one study, whereas another reported approximately 5% cell death at 10 µM, with a further increase at 20 µM [33,54]. Variations in stimulus, time, and assay make it impossible to define a uniform window. In addition, the predominance of assays based on extracellular DNA, Sytox Green staining, or microscopy, without complete colocalization analyses or adequate discrimination from apoptosis, secondary necrosis, and cell lysis, supports a reduction in the release of NET-associated structures but does not demonstrate selective inhibition of a specific NETosis pathway.

The relationship between celastrol exposure and neutrophil apoptosis also appears to depend on concentration and experimental stimulus. In lipopolysaccharide (LPS)-stimulated neutrophils, celastrol increased Bcl-2-associated X protein (Bax) and activated caspase-3 while reducing B-cell lymphoma 2 (Bcl-2), accompanied by lower TNF-α and IL-6 levels. However, limitations in cell-purity assessment and the unresolved fate of apoptotic neutrophils preclude determining whether these findings reflect selective removal of inflammatory cells or a broader cytotoxic effect [55]. Without efferocytosis assays, induction of apoptosis cannot be assumed to promote inflammatory resolution. An arginine-glycine-aspartic acid-modified bovine serum albumin formulation inhibited neutrophil NET formation, reduced citrullinated histone H3, and induced apoptosis in inflammatory neutrophils. However, it lacked exposure-matched comparisons with free celastrol, an empty carrier, and non-targeted nanoparticles [56].

Likewise, the in vivo improvement cannot be attributed specifically to neutrophils because the study did not use depletion, adoptive transfer, peptidyl arginine deiminase 4- or NOX2-deficient models, or rescue experiments. Chemotaxis, adhesion, degranulation, phagocytosis, antimicrobial activity, neutropenia, and bone marrow suppression remain poorly characterized. The relevance of celastrol in neutrophils from patients with RA, the specificity of its molecular targets, and the balance between immunomodulation and antimicrobial defense remain unresolved.

In vivo results may involve endothelial, myeloid, or stromal cells in addition to neutrophils. Reduced infiltration and cytokine levels in AIA or CIA are consistent with decreased neutrophil-associated inflammatory activity. However, these findings, by themselves, do not indicate that neutrophils are required for the therapeutic effect [55,56]. It also remains unclear whether concurrent modulation of NOX2 activity, ROS production, and NET-associated responses could affect antimicrobial host defense. Accordingly, the available evidence best supports acute modulation of selected neutrophil functions rather than coordinated or persistent reprogramming of the neutrophil compartment.

The interpretation of celastrol’s effects on neutrophils also depends on the activating stimulus, because pharmacological stimuli, TNF-α, immune complexes, and LPS engage partially distinct signaling modules [33,54,55]. Celastrol’s ability to reduce several outcomes across these conditions may therefore reflect modulation of a shared signaling node or a broader cellular perturbation. Responses to RA-derived IgG increase disease relevance [33]. However, neutrophils from healthy donors do not replicate the chronic exposure to hypoxia, cytokines, and the metabolic milieu of synovial fluid encountered in RA [53].

### 4.3. Dendritic Cells: Limited and Extrapolative Evidence

Evidence for celastrol in dendritic cells (DCs) in RA remains limited and largely indirect in RA, with available human data derived from monocyte-derived DCs from healthy donors rather than RA patients. In this system, 0.25 μM celastrol altered endosomal, lysosomal, and autophagic trafficking of fibrillar α-synuclein and reduced IL-1β, IL-6, and TNF-α mRNA expression, with a modest increase in IL-10. However, celastrol produced no substantial changes in major histocompatibility complex (MHC) class I or II, cluster of differentiation 80, or CD86. Peptide loading and cross-presentation were not directly assessed. In the same system, treated DCs reduced T helper 1 (Th1) and Th17 cell responses, but also decreased regulatory T-cell (Treg) frequency [57]. The pattern is compatible with lower immunostimulatory capacity, not stable tolerance. In experimental autoimmune encephalomyelitis (EAE), celastrol-pretreated bone marrow-derived dendritic cells (BMDCs) and splenic DCs isolated from celastrol-treated mice reduced Th17 responses in cocultures with naïve T cells. In a separate adoptive-transfer experiment, encephalitogenic lymphocytes—not treated DCs—were transferred, followed by systemic celastrol administration in recipient mice. Celastrol also reduced p65 phosphorylation in murine BMDCs, consistent with NF-κB pathway modulation; however, the necessity of this change for the subsequent reduction in Th17 responses was not established [58]. Comparable effects have not yet been demonstrated in DCs from patients with RA, in models using arthritogenic autoantigens, or in joint-relevant systems. Thus, DCs may represent a biologically plausible interface between innate and adaptive immunity, but their role as a celastrol-responsive population in RA remains insufficiently established.

Arthritis models provide only indirect evidence about DCs, as changes in Th1, Th17, and Treg cells were observed without direct DC isolation or functional evaluation [36,59]. In oncology, a celastrol hydrogel was associated with DC activation, a formulation- and microenvironment-dependent outcome that reinforces the lack of a uniform tolerogenic phenotype [60]. Citrullinated autoantigen uptake, peptide–MHC complex formation, cross-presentation, and autologous T-cell activation remain unstudied. Thus, DCs represent an extrapolative connection and an experimental priority, not an established target in RA.

### 4.4. Natural Killer (NK) Cells: Absence of Specific Evidence in RA

NK cells can exert inflammatory, regulatory, or osteoclastogenic functions depending on the compartment and activation state; therefore, their inhibition may not be beneficial in RA [61,62]. Direct evidence for celastrol derives from NK-92 cells studied outside an RA context, in which the compound was used as a pharmacological inhibitor of c-Myb and reduced cytotoxicity, interferon gamma (IFN-γ), natural killer group 2 member D (NKG2D), and granzyme B, while c-Myb silencing partially reproduced this phenotype [63]. The multitarget nature of celastrol precludes attributing the observed effect exclu-sively to c-Myb. Without data from primary circulating or synovial NK cells, the relevance of this population to celastrol-mediated anti-arthritic effects remains insufficiently defined.

NK cell relevance is complicated by their context-dependent functions, including IFN-γ production, RANKL- and M-CSF-associated activity, regulatory effects, and po-tential cytotoxicity against osteoclasts [61,62]. The study in NK-92 did not evaluate these functions or models of arthritis.

## 5. Adaptive Immunity

### 5.1. Axis Th1/IFN-γ: Limited Evidence

Evidence for celastrol-mediated modulation of the Th1/IFN-γ axis in RA remains limited and largely derives from systemic cytokine measurements and non-RA experimental systems. In rats with CIA, celastrol reduced serum IFN-γ, along with TNF-α, IL-1β, and IL-6, and was associated with lower NADPH oxidase 4-dependent oxidative stress [59]. The cellular origin of IFN-γ was not determined, and Th1-cell frequency, differentiation, and function were not evaluated. Outside of RA, celastrol decreased anti-CD3-induced Th1 cells among cluster of differentiation 4-positive (CD4+) T cells from MRL/lpr mice [64] and reduced specific responses by modifying antigen processing by DC [57,64]. These results are consistent with attenuation of the Th1/IFN-γ axis, but do not demonstrate that Th1 is a direct target in RA. Likewise, a reduction in systemic IFN-γ alone may not be sufficient to infer selective inhibition of Th1 differentiation.

Interpreting celastrol’s effects on the Th1 axis is further complicated by regulatory factors that have not been directly examined in celastrol-treated RA systems. T-bet and signal transducer and activator of transcription 4 (STAT4) regulate Th1 differentiation, and the G protein alpha q subunit (Gαq) may restrict this program. In RA patients, Gαq was inversely correlated with IFN-γ, but that study did not evaluate celastrol [65]. Similarly, meta-analyses of T. wilfordii preparations group extracts, triptolide, and celastrol, and do not allow attributing a collective decrease in Th1 to the isolated compound [66]. Demonstrating a T-cell-intrinsic effect requires studies using CD4+ T cells from patients with RA or from arthritic models, controlled polarization experiments, assessment of T-bet, STAT4, and IFN-γ, and evaluation of cell viability at pharmacologically plausible celastrol concentrations.

### 5.2. Axis Treg/Th17

Across arthritis models, celastrol has been associated with attenuation of Th17-related programs and increases in Treg-associated markers. In AIA, celastrol reduced synovial Th17 cells, increased Treg-associated markers, lowered the Th17/Treg ratio, and inhibited retinoic acid receptor-related orphan receptor gamma t (RORγt) and STAT3 signaling [36]. In separate murine T-cell polarization experiments, celastrol reduced glucose uptake and glycolysis-associated signaling in Th17 cells, including glucose transporter 1, hexokinase 2, mTOR, hypoxia-inducible factor 1 alpha (HIF-1α), c-Myc, and protein kinase B (AKT), while promoting fatty acid oxidation-associated signaling in induced Treg cells by increasing carnitine palmitoyltransferase 1A and the AMP-activated protein kinase alpha catalytic subunit [38]. These metabolic changes were accompanied by reduced Th17 cell generation and increased differentiation of forkhead box P3-positive (Foxp3^+^)-induced regulatory T cells, even though the metabolic experiments were conducted outside an arthritis model.

Additional mechanistic evidence from a non-RA inflammatory setting links celastrol to Th17 metabolic regulation via the HDAC3–FOXO3–GLS1 axis. In murine CD4^+^ T cells under Th17-polarizing conditions, celastrol inhibited HDAC3 activity, enhanced FOXO3-dependent repression of GLS1, reduced glutaminolysis, and limited Th17 differentiation. Functional perturbations, including GLS1 overexpression, FOXO3 silencing, and HDAC3 overexpression, supported dependence on this pathway, and biophysical and mutational experiments further supported a celastrol–HDAC3 interaction. HDAC3 overexpression also attenuated celastrol’s effects on Th17 responses and glutaminolysis in DSS-induced colitis. Thus, this axis has comparatively strong mechanistic support. However, its relevance to RA remains extrapolative because it has not been validated in arthritis models or in T cells from patients with RA [19].

Evidence for celastrol-associated dendritic cell–T-cell modulation in EAE comes from cocultures in which celastrol-pretreated bone marrow-derived DCs and splenic DCs isolated from celastrol-treated mice reduced Th17 responses in naïve T cells [58]. In a separate adoptive-transfer experiment, encephalitogenic lymphocytes were transferred, followed by systemic celastrol administration in recipient mice; treated DCs were not transferred. Together, these findings support directional functional modulation, but potential residual celastrol exposure during coculture and the absence of DC-specific necessity or rescue experiments limit causal attribution. Their relevance to RA remains extrapolative.

A broader multicompartment interpretation is supported by effects observed across myeloid and stromal systems. A silver-modified ceria nanoplatform loaded with celastrol increased Foxp3-positive, cluster of differentiation 25-expressing cells, reduced IL-1β, IL-6, and TNF-α, and was associated with a more favorable joint inflammatory microenvironment than either free celastrol or the drug-free carrier [67]. Additional studies have reported modulation of macrophage inflammatory phenotypes, neutrophil effector responses, and FLS signaling and survival pathways [27,28,32,55]. Collectively, these findings suggest a less Th17-supportive inflammatory environment, although they primarily reflect convergent effects across compartments rather than direct intercellular dependence. Carrier-related contributions also remain relevant in formulation-based studies [67].

Extrapolative evidence from lupus models further supports Treg/Th17 modulation, with increased Treg responses and reduced autoreactive T-cell activity reported outside RA [64,68]. Differences in disease context and outcome quantification complicate synthesis across inflammatory models, including EAE [58,67]. Formulation-based studies introduce an additional source of uncertainty because carrier contributions and the effective amount of celastrol released may not be well defined [67]. Changes in the Treg/Th17 ratio may reflect altered survival, redistribution, proliferation, or differentiation, so frequency alone cannot distinguish these possibilities from functional suppression. Overall, convergence across AIA, EAE, lupus, and other inflammatory models increases mechanistic plausibility, while direct confirmation in human RA remains limited.

### 5.3. B lymphocytes: COMMD3/8, Cell Trafficking and Humoral Response

B lymphocytes contribute to RA through autoantibodies, antigen presentation, co-stimulation, and cytokines. The available evidence does not support attributing celastrol’s overall efficacy to direct, broad modulation of this population. The main exception is the COMMD3/8 complex, an adapter required for responses to chemoattractant receptors. Celastrol covalently bound to COMMD3, destabilized the heterodimer, restricted B-cell migration, and reduced humoral responses and arthritis in mice. A resistant variant abolished disruption and the therapeutic effect, establishing a rare causal link between molecular interaction, cell function, and in vivo efficacy [17]. This result does not demonstrate that celastrol directly inhibits proliferation, differentiation, or antibody production across all B-cell subpopulations. Its differential effects on regulatory, memory, germinal center, and plasma cells, as well as on synovial ectopic lymphoid structures, remain uncertain. It has also not been established whether COMMD3/8 disruption accounts for a dominant proportion of RA activity or whether it converges with extrinsic niche changes.

The reduction in autoantibodies provides functional evidence of humoral suppression, although the underlying mechanism remains unclear [17,69]. The included studies did not systematically evaluate rheumatoid factor, anti-citrullinated protein antibodies, isotype switching, affinity, epitope specificity, immune-complex formation, or parallel B-cell subpopulations. Signaling through B-cell activating factor (BAFF) and its receptor, B-cell activating factor receptor (BAFF-R), is biologically plausible as a mechanism supporting B-cell survival and differentiation in RA. However, celastrol has not been shown to directly modulate this axis in B lymphocytes. BAFF, its receptors, and NF-κB were not assessed in celastrol-treated B cells, and findings from Janus kinase–STAT signaling or phosphoinositide 3-kinase (PI3K)/AKT/mTOR signaling in other cell types cannot be assumed to apply directly to B cells. Studies examining BAFF, plasma cells, or Th17 responses outside B cells provide additional context [70,71,72]. However, overall, the evidence more clearly supports humoral suppression than repertoire reprogramming or a defined B-cell-intrinsic mechanism.

Extrinsic mechanisms may also contribute to B-cell persistence and function in the synovial environment. Celastrol attenuates selected myeloid and stromal signals, reduces NET-associated responses, and modifies FLS behavior. Independently, TNF-α and BAFF have been shown to enhance B-cell adhesion to FLS via human vascular cell adhesion molecule 1 [73]. Celastrol also reduces humoral responses [17] and, separately, suppresses RA IgG-induced NET formation [33], raising the possibility that it may influence both B-cell–stromal support and an antibody–neutrophil axis. Taken together, these observations support mechanistic convergence across B-cell, stromal, and neutrophil compartments, although the directionality, mediators, and functional dependence of these proposed intercellular links remain to be clarified.

Evidence from lupus models also suggests that reduced humoral responses may partly reflect effects outside the B-cell compartment, including the elimination of autoreactive T cells [64], although the relevance of this mechanism to RA remains uncertain. In contrast, loss of celastrol responsiveness in the resistant mutant provides functional evidence linking COMMD3/8 engagement to altered B-cell migration and to the therapeutic response observed in the model [17]. However, this finding alone does not indicate that all humoral responses or effects in other cellular compartments depend on this complex. The lack of systematic data on B-cell subpopulations, B-cell receptor, CD40/CD40 ligand, Toll-like receptor signaling, plasma-cell differentiation, the circulating and synovial compartments, and serological subgroups further limits broader interpretation. Thus, COMMD3/8 provides a relatively strong mechanistic anchor, but its role across the broader B-cell compartment and its potential value as a biomarker still require additional validation.

Celastrol’s effects on B-cell trafficking do not necessarily imply a restoration of immune tolerance. Restricting B-cell migration may reduce access to supportive niches and decrease antibody responses without necessarily altering memory, affinity maturation, or intrinsic autoreactivity [17]. Even if these effects prove reversible after treatment withdrawal, the mechanism could remain pharmacologically useful without implying durable re-establishment of tolerance. Withdrawal and rechallenge studies would therefore be particularly informative for distinguishing transient control of B-cell trafficking from more persistent changes in the compartment.

## 6. Fibroblast-like Synoviocytes (FLSs) in the Rheumatoid Synovium

FLS are major contributors to synovial hyperplasia, leukocyte recruitment, extracellular matrix degradation, and bone erosion in RA [2,3]. Across studies of rheumatoid arthritis fibroblast-like synoviocytes (RA-FLSs), celastrol has most consistently been associated with reduced proliferation, migration, invasion, and inflammatory mediator production. In primary RA-FLS, celastrol reduced viability and induced G2/M cell-cycle arrest, DNA damage, and apoptosis, accompanied by an increased Bax/Bcl-2 ratio, cleavage of caspase-3, caspase-9, and poly(ADP-ribose) polymerase, and reduced Fas receptor expression [41]. Increased lactate dehydrogenase release indicates that cytotoxicity contributes to at least part of this response. Because few studies have compared RA-FLS with non-RA fibroblasts or with lining and sublining fibroblast subsets, the relative contributions of functional modulation and cell depletion, as well as any preferential effect on pathogenic FLS populations, remain incompletely defined.

At concentrations described as non-cytotoxic, celastrol restricted migration and invasion and reduced matrix metalloproteinase-9 (MMP-9) expression in interleukin-17A (IL-17A)-stimulated human FLS [31]. In synovial fibroblasts from arthritic rats, celastrol reduced Mtb-induced IL-6 production and MMP-9 activity and decreased pERK, whereas migration and invasion were not evaluated in that experimental system [69]. Transcriptomic analysis of RA-FLS further identified changes in CCL2, CXCL10, CXCL12, CCR2, and CXCR4 expression. These changes were accompanied by reduced protein levels of IL-6, IL-8, and monocyte chemoattractant protein 1 (CCL2) [29]. NF-κB is the most consistently observed functional signaling node, with studies reporting inhibition of IκBα phosphorylation, p65 nuclear translocation, promoter occupancy, and MMP-9 transcription [29,31]. Together, these findings are consistent with attenuation of the migratory, invasive, and inflammatory functions of FLS. However, NF-κB appears to act primarily as a downstream signaling node, and the findings for MMP-9 may not necessarily generalize to other metalloproteinases.

Several mechanistic studies converge on mTOR regulation, although the strength of evidence varies across upstream pathways. Celastrol inhibited sarco/endoplasmic reticulum Ca^2+^-ATPase, increased cytosolic Ca^2^^+^, and activated calcium/calmodulin-dependent protein kinase kinase beta (CaMKKβ)/AMP-activated protein kinase (AMPK) signaling, resulting in mTOR inhibition and autophagy-dependent cell death. Rescue with 1,2-bis(o-aminophenoxy)ethane-N,N,N’,N’-tetraacetic acid acetoxymethyl ester (BAPTA/AM) and silencing of Ca^2+^-dependent proteins further strengthened this functional relationship [27]. A separate study linked PI3K/AKT/mTOR inhibition to changes in the microtubule-associated protein 1 light chain 3-II (LC3-II) to- LC3-I ratio, sequestosome 1 (p62), and Beclin-1, along with reduced proliferation [37]. In parallel, biophysical and genetic evidence supports binding to IGF2BP3 and inhibition of the IGF2BP3/RASGRF1–mTORC1 axis [18]. Thus, mTOR emerges as a recurrent point of mechanistic convergence, but the degree of validation of the upstream pathways is not equivalent.

Additional pathways broaden the mechanistic landscape of celastrol in RA-FLS. In TNF-α-stimulated FLS, celastrol reduced NLRP3, ASC, and cleaved caspase-1 and decreased the amount of NLRP3 coimmunoprecipitated with Hsp90 [26]. Although Octet RED96 measurements suggested a celastrol–Hsp90 association, the reported binding affinity is difficult to reconcile with the nanomolar assay concentrations, and the concomitant reduction in total NLRP3 prevents definitive attribution of the coimmunoprecipitation result to direct disruption of the Hsp90–NLRP3 complex. Separately, reduced migration and invasion under hypoxic conditions were accompanied by decreased HIF-1α/CXCR4 signaling [74]. Celastrol has also been associated with increased susceptibility to ferroptosis, accompanied by changes in prostaglandin-endoperoxide synthase 2 (PTGS2), iron accumulation, lipid peroxidation, and mitochondrial function, with partial attenuation of celastrol responses following PTGS2 overexpression [75]. A separate study linked celastrol to activation of the nuclear factor erythroid 2-related factor 2 (Nrf2)/heme oxygenase-1 (HO-1)/glutathione peroxidase 4 (GPX4) pathway in TNF-α-stimulated FLS and in experimental arthritis, with pharmacological activation and inhibition of this pathway modifying selected celastrol-associated responses [40]. These findings support pathway involvement but do not establish Nrf2, HO-1, or GPX4 as direct molecular targets of celastrol. Transcriptomic changes in inflammatory and chemokine-related genes [29], together with apoptosis [41], autophagy-dependent cell death [27], and ferroptosis-associated responses [40,75], suggest that signaling modulation, cellular stress, and multiple routes of cell elimination may coexist and vary with experimental context.

Multicellular models extend these observations beyond isolated FLS. A hydrogel containing celastrol-loaded nanomedicine attenuated proinflammatory macrophage features and fibroblast proliferation, while a dual strategy targeting macrophages and FLS reduced synovial hyperplasia [12,76]. A chip incorporating SW982 cells, osteoclasts, and osteogenic cells showed reduced fibroblast migration and tartrate-resistant acid phosphatase (TRAP) activity [77]. Synovium-on-a-chip systems containing RA-FLS, macrophages, and endothelium preserved multicellular interactions [11]. These systems support celastrol activity in complex cellular environments, but their aggregate readouts offer limited resolution for assigning directional paracrine signals, identifying necessary mediators, or distinguishing carrier-specific contributions. Translational interpretation also depends on ex vivo exposure, cellular heterogeneity, dose–response relationships, and controls that separate signaling effects from cytotoxicity. Overall, the available evidence supports inhibition of pathogenic FLS functions and, in some settings, depletion of activated cells, rather than demonstrating stable stromal reprogramming. Small human samples, differences between cell lines and primary cells, and limited follow-up further constrain extrapolation.

## 7. Osteoclasts, Immunostromal Signals, and Structural Protection

Osteoclasts are the primary effector cells responsible for bone erosion in RA, although RANKL, inflammatory cytokines, and stromal signals shape their differentiation and activity. Celastrol may therefore influence bone resorption both directly, through effects on osteoclast precursors, and indirectly, by modifying osteoclastogenic signals [6,7,34,39,44]. In bone marrow-derived macrophages and RAW264.7 cells stimulated with M-CSF/RANKL, nanomolar concentrations of celastrol reduced TRAP-positive multinucleated cells, F-actin ring formation, and resorption on dentine slices and decreased expression of TRAP, cathepsin K, calcitonin receptor, dendritic cell-specific transmembrane protein, ATPase H+ transporting V0 subunit d2, and vacuolar H+-ATPase subunit a3 [34]. Reduced osteoclast and cathepsin K-positive cell numbers in arthritic joints further support the in vivo relevance [6,44]. However, apoptosis was observed in some experimental systems, and viability controls were heterogeneous, making it difficult to fully distinguish inhibition of osteoclast differentiation from cytotoxic depletion.

Mechanistic evidence involves both direct regulation of osteoclastogenic signaling and changes in the surrounding inflammatory environment. Celastrol reduced signaling along the RANKL–TAK1–NF-κB/MAPK–c-Fos/NFATc1 axis, including phosphorylation of transforming growth factor beta-activated kinase 1 (TAK1), IκBα/p65, and MAPKs, as well as downstream expression of c-Fos and NFATc1 [34]. In parallel, lower RANKL, increased osteoprotegerin (OPG), a reduced RANKL/OPG ratio, and decreases in IL-1β, IL-6, interleukin-17 (IL-17), IL-18, and TNF-α were reported in AIA [78]. Together, these findings are consistent with attenuation of osteoclastogenic signaling and reduced osteoclastogenic pressure originating from immune and stromal compartments. However, the available studies do not identify a necessary molecular target or clarify the relative contributions of individual cellular sources.

Evidence of structural protection in vivo primarily derives from experimental arthritis models. In AIA, celastrol preserved joint architecture, reduced osteoclast numbers at erosion sites, and improved bone-related structural outcomes [6,44]. In CIA, celastrol formulations attenuated synovitis, cartilage degradation, and bone erosion, and improved structural imaging parameters [28,50,67]. In the folate-modified liposome study, folate-modified celastrol liposomes showed greater preservation of joint architecture than free celastrol or non-targeted liposomes across micro-CT, MRI, and histological assessments [50]. These findings support formulation-associated structural protection but do not establish a direct osteoclast mechanism. More recently, free celastrol was also associated with reduced joint destruction, preservation of cartilage-related histological features, lower osteoclast-associated staining, and modulation of the OPG/RANKL balance in CIA [40]. These findings extend the structural evidence for free celastrol but do not establish whether the bone effects arise from direct osteoclast inhibition, altered stromal or immune signaling, or broader suppression of synovial inflammation. Titanium-induced osteolysis and ovariectomy-associated bone loss models further support anti-osteoclastic activity outside synovitis, although their relevance to RA is more limited [34].

Interpretation of the structural findings depends in part on the timing of celastrol administration. Preventive designs interfere with early osteoclastogenesis, whereas therapeutic designs can help prevent further erosive damage; neither design alone repairs established structural lesions [44]. Post-treatment follow-up is also needed to determine whether structural benefits persist after celastrol withdrawal.

Evidence from experimental systems suggests that celastrol’s effects on bone remodeling may extend beyond osteoclast suppression. In AIA, decreases in osteoclasts and osteoblasts were accompanied by reductions in resorption markers and procollagen type I N-terminal propeptide. In contrast, selected experimental systems showed inhibited osteoblast growth, differentiation, and nodule formation [44,79]. This pattern could reflect normalization of accelerated turnover or broader suppression of remodeling, a distinction that would require dynamic histomorphometry and post-treatment assessment. Translational interpretation is further constrained by the narrow separation between efficacy and toxicity, as the lowest effective oral dose in AIA was close to doses associated with thymic or hepatic alterations and mortality [44]. Additional limitations include the lack of human osteoclast data, active comparators, consistent reporting of randomization and blinding, and the need for prolonged evaluation of bone remodeling.

Overall, celastrol inhibits osteoclast differentiation and function and is associated with preclinical structural protection. In contrast, the relative contributions of direct osteoclast effects, broader osteoimmune modulation, and changes in bone formation remain context-dependent. The available evidence therefore supports preclinical bone-protective activity, but durable, clinically validated structural disease modification has not been established. Taken together, these studies reveal uneven depth and directness of evidence across immune, stromal, and osteoimmune compartments relevant to RA (Table 2). Figure 2 summarizes the cellular evidence landscape and the uneven strength of the supporting experimental evidence.

## 8. Multicellular Convergence: Evidence and Limitations of Crosstalk

A key issue in interpreting celastrol’s multicellular effects is whether changes across cell populations reflect independent or convergent responses, or true modulation of the intercellular circuits that sustain RA pathology. Much of the available evidence derives from observations in individual cell populations or from concurrent changes measured at the tissue level. Shared modulation of NF-κB-, MAPK-, mTOR-, or ROS-related pathways therefore supports multicellular convergence but does not, by itself, demonstrate causal intercellular communication.

### 8.1. Myeloid–Stromal Interface

The macrophage–FLS axis is among the best-supported multicellular interactions in RA. Celastrol reduces inflammatory programs and CD68^+^ macrophage abundance and, in separate studies, inhibits FLS proliferation, chemokine production, migration, and invasion [29,31,45]. Shared inhibition of NF-κB across both populations therefore supports pharmacological convergence, but this alone does not demonstrate causal crosstalk. A synovium-on-a-chip model incorporating RA-FLS, M1 macrophages, and endothelial cells supported coordinated celastrol activity under sustained multicellular inflammatory conditions [11]. Meanwhile, a hydrogel containing celastrol-loaded nanomedicine reduced M1-associated features and FLS proliferation in CIA [12]. These studies support multicellular activity and provide complementary evidence from conditioned-medium experiments. However, the available findings do not establish a necessary intercellular mediator or fully distinguish cell-derived signaling effects from potential contributions of residual celastrol or the delivery system. Consequently, directional and causal macrophage–FLS dependence remains insufficiently established. In formulation-based systems, interpretation must also account for contributions from local release, hyaluronic acid, biodistribution, and other carrier-related effects [12], as well as the potential biological contribution of hyaluronic acid [80].

Candidate mediators of macrophage–FLS communication can be prioritized based on experimentally observed changes. Celastrol modulates FLS-derived chemokines and inflammatory signaling, as well as myeloid cytokine and redox responses [29,31,45]. Integrated macrophage–FLS systems further support the simultaneous modulation of both compartments but have not identified a necessary mediator [11,12]. Establishing such dependence would require resolving the emitted signal, the corresponding receptor, and the downstream response using approaches such as conditioned-medium or contact-separation experiments, selective perturbation of the proposed sender population, blockade of the mediator or receptor, and functional rescue.

Celastrol has been shown to suppress neutrophil inflammatory responses relevant to the synovial environment, although its downstream effects on stromal cells have not been directly demonstrated. Celastrol inhibited ROS- and NET-associated responses in RA IgG-stimulated neutrophils [33]. However, these changes were not subsequently linked to reduced FLS activation, antigen presentation, or osteoclastogenesis. The parallel suppression of ROS- and NF-κB-related responses across neutrophils, macrophages, and FLS therefore supports multicellular convergence rather than indicating disruption of a neutrophil–stromal circuit.

### 8.2. DC–T Interface and Adaptive Immune Convergence

Evidence for celastrol-associated directional DC–T-cell modulation primarily comes from non-RA models. In EAE, celastrol-pretreated DCs and splenic DCs isolated from celastrol-treated mice reduced Th17 responses in subsequent cocultures with naïve T cells [58]. A separate adoptive-transfer experiment used encephalitogenic lymphocytes followed by systemic celastrol administration, rather than transferring treated DCs. In a human α-synuclein model using cells from healthy donors, celastrol-pretreated monocyte-derived DCs reduced antigen-specific Th1 and Th17 responses alongside a reduction in Treg frequency [57]. Together, these findings support directional functional modulation in coculture. However, potential residual celastrol exposure, the lack of DC-specific necessity or rescue experiments, and the use of non-RA models limit causal attribution and extrapolation to RA. 

Celastrol has been shown to modulate Th17-related responses and, in separate experiments, to attenuate IL-17A-driven behavior in FLS, though these effects are not directly linked. In AIA, celastrol reduced Th17 responses and increased Foxp3-associated markers [36], while separate experiments showed inhibition of migration and invasion in IL-17A-stimulated human FLS [31]. These findings are consistent with modulation of both the cytokine-producing and cytokine-responsive compartments, but no Th17–FLS coculture, conditioned-medium experiment, IL-17/IL-17R blockade, or rescue study has shown that celastrol alters transmission across this circuit.

Celastrol also affects B-cell and neutrophil responses in ways that could converge functionally, although the intercellular sequence linking these effects has not been demonstrated. COMMD3/8 provides relatively strong evidence for a B-cell-intrinsic effect of celastrol [17], whereas separate studies show suppression of IgG-induced neutrophil ROS and NET-associated responses [33]. However, antibodies generated during celastrol treatment have not been functionally tested for their ability to form immune complexes or activate neutrophils, and direct modification of B-cell–FLS communication has not been demonstrated. Thus, reduced humoral responses and neutrophil effector activity support multicompartment convergence, but the proposed B-cell→antibody→neutrophil sequence and B-cell–FLS interaction remain untested as causal intercellular pathways.

### 8.3. Osteoimmunological Convergence and Bone Remodeling

Evidence for stromal–osteoclast convergence derives from multicellular models rather than from direct demonstration of a necessary intercellular signal. A microfluidic chip integrating SW982 cells, RAW264.7-derived osteoclasts, and osteogenic cells showed simultaneous reductions in fibroblast migration and TRAP activity during celastrol treatment [77], complementing the direct anti-osteoclastogenic effects described in separate systems [34]. This model supports activity within a multicellular erosion module. However, its use of cell lines and the absence of ligand–receptor blockade or population-specific perturbation prevent the identification of a necessary FLS–osteoclast pathway.

In vivo, concomitant reductions in macrophages, osteoclasts, inflammation, and structural damage suggest that both direct and indirect mechanisms may contribute to reduced bone resorption. However, these studies cannot determine the relative contributions of these mechanisms [6,45]. An MMP-9-responsive, RGD-modified celastrol nanoparticle system targeted inflammatory macrophages and osteoclasts and was associated with reduced inflammation and bone erosion in advanced AIA, with complementary uptake and apoptosis assays in cells from patients with RA [48]. However, these findings support multicellular targeting by the compound–carrier system rather than causal communication between macrophages and osteoclasts. Changes in osteoblast-related outcomes further caution against equating structural protection with normalization of bone remodeling [44,79]. Likewise, without experiments that separate precursor-intrinsic effects from stromal and immune-cell contributions, such as conditioned-medium approaches, selective perturbation, or mediator blockade, changes in RANKL and OPG cannot be attributed to a specific cellular source. Table 3 summarizes the highest level of evidence for the principal cellular circuits evaluated with celastrol.

### 8.4. Hierarchy of Evidence and Limits of Multicellular Inference

Interpreting the multicellular effects of celastrol requires distinguishing the active compound’s contribution from that of the delivery system and avoiding overinterpretation of phenotypic readouts. Formulation-based findings require attribution to the compound–carrier system when carrier-related effects cannot be excluded [28,49,67]. Changes in phenotypic markers, cell frequency, or viability alone do not demonstrate stable functional reprogramming [27,34,36,39,40,41,52,81,82,83,84,85,86]. The maximum evidence level assigned to each proposed cellular circuit (Table 3) was determined by the experimental design and the strength of the demonstrated intercellular relationship, rather than by the number of concurrently affected cell populations. Directional functional evidence (Level 4) was assigned to experimental designs that assessed the influence of a celastrol-conditioned cell population on another. Potential residual drug exposure and the absence of cell-specific perturbation or rescue were considered limitations for causal attribution. Persistent reprogramming (Level 5) additionally required evidence of sustained intercellular effects after celastrol withdrawal.

Overall, the available evidence predominantly supports separate cellular effects, mechanistic convergence, and activity within multicellular systems (Levels 1–3) [11,12,17,29,31,33,34,36,48,77]. The DC–T/Th17 relationship was assigned Level 4 based on directional functional evidence from cocultures of celastrol-conditioned DCs and T cells. However, this evidence derives from non-RA systems, and residual drug exposure and DC-specific causal necessity remain unresolved [57,58]. No evaluated circuit reaches Level 5, because persistence of the altered intercellular interaction after celastrol withdrawal has not been demonstrated. Figure 3 summarizes this hierarchy and the maximum level reached by each evaluated intercellular relationship.

## 9. Limitations of the Evidence

The predominantly preclinical nature of the available evidence limits how far findings on celastrol can be extrapolated to the heterogeneity of human RA. Most studies rely on CIA or AIA models, immortalized cell lines, or relatively small primary-cell samples. These systems are useful for demonstrating pharmacological activity and exploring mechanisms, but they capture only part of the disease’s immune, stromal, and temporal complexity [87,88]. Direct human evidence is primarily found in FLS and in selected experiments using cells from healthy donors [29,33,57]. Small donor numbers further limit the representation of interindividual and fibroblast-subpopulation heterogeneity [89,90], while commonly used cell lines may differ from primary cells in proliferation, metabolism, and stress responses [91,92]. As a narrative-review observation, confidence is further reduced when studies have small sample sizes or incompletely report randomization, blinding, statistical power, or cellular quality control. The Animal Research Reporting of In Vivo Experiments 2.0 guidelines identify several of these elements as relevant reporting standards for animal research [93]. In some settings, inadequate characterization of cell purity may also affect functional readouts, as illustrated by the potential contribution of minor monocyte contamination to neutrophil cytokine measurements [94]. Independent replication remains limited, and substantial variation in models, doses, and formulations complicates direct comparisons across studies [28,36,44]. These limitations do not negate celastrol’s reported activity, but they reduce confidence in its magnitude and generalizability.

Mechanistic confidence also varies substantially across the available evidence. Phenotypic or signaling changes alone do not establish a primary molecular target or causal intercellular dependence [25]. COMMD3/8 provides the most complete molecular–functional validation, whereas IGF2BP3 has biophysical and genetic support that still requires independent replication [17,18]. HDAC3 is supported by biophysical, cellular target-engagement, and functional/mutational evidence, but only in non-RA T-cell and colitis systems [19]. IKKβ has strong biochemical and mutational support centered on Cys179, although direct adduct identification, cellular target engagement, and RA-specific validation remain lacking [20]; for p47phox/NOX2, biochemical and cellular evidence supports the proposed mechanism, although cellular target engagement and relevance to anti-arthritic efficacy remain unresolved [21]. Most other proposed signaling nodes remain supported mainly by downstream or associative evidence. This uneven mechanistic depth is particularly relevant for an electrophilic compound with potentially broad protein reactivity at higher exposures [95,96,97]. As summarized in Table 3, most RA-relevant intercellular circuits are supported by mechanistic convergence or activity in multicellular systems rather than demonstrated directional dependence. In contrast, the strongest directional evidence derives from non-RA DC–T/Th17 models [11,12,48,57,58,77]. 

Even when preclinical activity is reproducible, its translational significance depends on whether effective responses can be achieved at relevant and tolerable exposures. In vitro concentrations in the nanomolar-to-micromolar range may overlap with cytotoxic effects and are rarely directly related to free concentrations attainable in plasma or synovial tissue [27]. Unfavorable and variable exposure, together with a narrow preclinical therapeutic window, limits translation of the reported cellular effects [9,10,13,14,42,44]. Broader formulation and delivery challenges have been reviewed elsewhere [46]. Formulation studies often lack sufficient controls or exposure measurements to isolate celastrol’s contribution [28,43,48,50,56]. Short follow-up and predominantly preventive or early-treatment designs further limit assessment of sustained efficacy and chronic toxicity. Accordingly, clinical efficacy, dose–exposure–response relationships, and clinically acceptable safety for celastrol in RA remain undefined. These preclinical safety concerns substantially constrain development of the free compound, although the available evidence does not establish why clinical studies of celastrol in RA have not been undertaken. Current evidence is therefore insufficient to support purified celastrol in its present form as a clinically viable RA therapy.

Taken together, these constraints require weighting the evidence by experimental relevance and the depth of validation rather than treating it as a uniform body of support. FLS, macrophages, and osteoclasts have a more direct experimental basis for RA, whereas evidence for DCs, NK cells, and some T-cell mechanisms relies more heavily on extrapolation [27,34,48,57,58,63]. A validated molecular interaction, a phenotypic change, an effect observed with a complex formulation, and a mechanism derived from another disease context should therefore not carry equal interpretive weight [17,25,28]. Several mechanistically informative findings derive from non-RA inflammatory or bone-loss models and therefore require disease-specific validation [34,57,58,60,64]. The same distinction applies to *Tripterygium wilfordii* preparations and related compounds, which may provide relevant pharmacological context but do not constitute direct evidence for purified celastrol. 

## 10. Validation and Development Priorities

### 10.1. Define Causal Pharmacology and Selectivity

Stronger mechanistic attribution requires orthogonal binding assays, cellular target-engagement measurements, and functional perturbation at active but non-cytotoxic exposures. These approaches should prioritize independent validation of IGF2BP3 in human RA cells and RA-specific evaluation of the HDAC3–FOXO3–GLS1 mechanism, while using COMMD3/8 as the most firmly supported molecular–functional benchmark [17,18,19]. Where applicable, mutational, loss-of-function, resistance, and rescue experiments should determine whether target engagement is necessary for the corresponding cellular and anti-arthritic effects. Proteome-wide profiling could further distinguish reproducible polypharmacology from broad thiol modification.

Structure–activity studies should determine whether derivatives or prodrugs preserve target engagement and validated function while widening the efficacy-toxicity window. Because covalent reactivity appears to contribute to at least one well-supported mechanism involving COMMD3/8, reducing electrophilicity may not necessarily improve the overall pharmacological profile [17]. A more informative objective is to retain validated mechanisms while reducing nonspecific proteome reactivity and systemic toxicity.

### 10.2. Integrating Exposure, Efficacy and Safety

Translational interpretation requires linking the administered dose to free celastrol concentrations, Cmax, AUC, metabolites, synovial exposure, target engagement, functional effects, efficacy, and toxicity. In vitro concentrations are difficult to interpret unless linked to joint exposure, particularly given celastrol’s poor and variable oral bioavailability [9,10] and its extensive metabolism [42]. The narrow separation between effective and toxic exposures reported preclinically further constrains translational interpretation [44]. Serial sampling in repeated-dose studies would help determine whether target engagement precedes functional change, inflammatory improvement, and structural protection, while post-withdrawal follow-up would clarify persistence.

A more informative safety assessment would consider whether suppression of pathogenic biomarkers is accompanied by preservation of protective immune and tissue functions. Evaluate reduced ROS- and NET-associated responses alongside antimicrobial defense; macrophage modulation alongside phagocytosis, efferocytosis, and pro-resolving functions; increased Treg measures alongside suppressive capacity; and bone protection alongside osteoclast–osteoblast coupling. The therapeutic window is better defined by exposure than by nominal dose because absorption, protein binding, metabolism, and tissue accumulation can yield different free concentrations at the same administered dose. Comparisons between free celastrol and formulations would be more informative if plasma and joint exposure were normalized whenever possible, allowing reductions in toxicity to be interpreted more confidently as reflecting lower systemic exposure, improved targeting, or carrier-related effects [28,48,56]. A recent oral self-microemulsifying formulation demonstrated greater efficacy than free celastrol at the same nominal dose, while a separate repeated-dose experiment suggested an improved biochemical safety profile [43]. However, the authors did not quantify plasma or joint celastrol exposure, so these findings do not support firm conclusions about improved bioavailability or a broader exposure-defined therapeutic window.

Repeated-exposure studies need to characterize organ- and system-level risk beyond short-term serum chemistry and histology. Assessing renal and hepatic function, hematology, susceptibility to infection, and, when relevant, reproductive and cardiovascular toxicity would provide a more informative safety profile. The absence of short-term abnormalities alone is insufficient to establish safety for further clinical development, particularly given the proximity of efficacious and toxic exposures reported for celastrol [8,44].

### 10.3. Prioritize Established Disease and Human Validation

Greater disease relevance would come from complementing preventive studies with treatment of established arthritis and post-treatment follow-up. These designs would help distinguish control of active inflammation, prevention of new erosions, reversal of synovitis, and persistence after withdrawal. Existing studies vary in treatment timing and, on their own, do not demonstrate repair of established structural damage [6,44,45]. Comparisons with free celastrol, vehicle, and standard therapy would provide a clearer baseline before evaluating combination regimens. Methotrexate combinations would be more interpretable once monotherapy is characterized and genuine dose- or toxicity-sparing effects are demonstrated. Post-treatment follow-up would also help determine relapse, erosion progression, and recovery of immune and bone function. Human validation would be more informative if it extended beyond small or narrowly selected cell samples to include blood, synovial fluid, and synovial tissue from patients characterized by disease activity, stage, serostatus, and treatment. At pharmacologically plausible exposures, such studies could evaluate COMMD3/8-dependent B-cell migration [17], neutrophil functions [33], Treg/Th17 programs [36], myeloid states [47], FLS responses [29], and osteoclastogenesis [34]. Greater confidence in a mechanism would require concordance among exposure, target engagement, and functional response. In contrast, effects dominated by loss of viability would weigh against further development of the free compound. Reproducibility across donors is better treated as an empirical outcome rather than assumed, given the cellular and molecular heterogeneity of RA synovium [88,89]. Restricted responses may identify biomarkers, whereas broad suppression accompanied by reduced viability would more strongly suggest nonspecific toxicity. Viability controls, appropriate non-RA cells, and cross-compartment comparisons would provide a stronger basis for distinguishing selective pharmacological effects from generalized toxicity.

### 10.4. Proving Multicellular Causation

Demonstrating multicellular causation requires experimental systems that maintain a common, quantified celastrol exposure while enabling cell-specific manipulation. Primary-cell cocultures, conditioned-medium experiments, contact-separation systems, and synovium-on-a-chip platforms could be combined with selective depletion or perturbation of the proposed sender population, neutralization of candidate mediators, receptor blockade, and functional rescue. These approaches are particularly relevant to the macrophage–FLS [11,12], DC–T/Th17 [57,58], Th17–FLS [31,36], B-cell–antibody–neutrophil [17,33], and FLS–osteoclast [34,77] circuits.

Temporal and spatial sampling should establish the sequence of cellular responses and determine whether perturbing one compartment alters the response of another. A causal circuit would be supported by loss of the intercellular effect after cell- or mediator-specific blockade and its restoration through rescue. Washout and rechallenge experiments would further distinguish reversible suppression during celastrol exposure from persistent alteration of the intercellular interaction.

### 10.5. Define Development Milestones and Stopping Criteria for Formulations

Formulation development is more reliably evaluated using controls that distinguish carrier-related effects from celastrol pharmacology. Each platform is best assessed against an empty carrier, free celastrol at a matched dose and, where feasible, at a matched exposure, a non-targeted formulation, and the loaded platform [12,28,48,56]. Recent formulation studies partially illustrate this framework. Folate-targeted liposomes incorporated free celastrol, and non-targeted comparators were used alongside folate-competition experiments [50]. In contrast, one study compared an oral self-microemulsifying formulation with free celastrol at the same nominal dose and included an empty-carrier control in the macrophage cytotoxicity assay [43]. However, neither study provides quantitative plasma and joint celastrol exposure data sufficient for exposure-matched attribution, so superior nominal efficacy should not be interpreted as evidence of improved bioavailability or targeting. Stability evidence is formulation- and assay-specific, encompassing ROS-responsive micelle behavior [28], hydrogel degradation and in vitro release [12], short-term serum stability [48], refrigerated liposome storage [50], longer-term monitoring of droplet properties and drug content [43], and nanoparticle stability under defined PBS and serum-containing conditions [56]. These measurements should not be interpreted as equivalent evidence of prolonged in vivo drug stability. Empty-carrier controls also differ in scope: unloaded micelles and BSA–RGD nanoparticles were assessed in selected cellular or animal experiments [28,56], whereas other studies evaluated separate hydrogel components [12], restricted empty-carrier testing mainly to viability or hemolysis [43,50], or lacked a matched unloaded carrier in their principal efficacy comparisons [48]. 

Progression toward clinical development would be more convincingly supported by a reproducible RA-relevant mechanism, achievable effective exposure, separation of efficacy from toxicity, benefit in established disease, and human validation, rather than by superior nominal efficacy alone. These milestones can also guide discontinuation decisions when key translational criteria are not met. If benefit occurs only at cytotoxic exposures, disappears after exposure matching, or is not reproduced in human systems, further development of free celastrol would be difficult to justify. A formulation with superior joint exposure, clearer mechanistic attribution, and improved safety would be more appropriately regarded as a compound–carrier product rather than as general validation of celastrol. A stepwise approach could assess mechanism and exposure in human systems, therapeutic and structural effects in established disease, and preservation of protective immune and bone-remodeling functions before further development. Such evidence could help define whether celastrol is better suited as a therapeutic candidate, a pharmacological probe, or a scaffold for safer derivatives.

## 11. Conclusions

Celastrol shows consistent preclinical activity across multiple components of rheumatoid arthritis, including myeloid cells, lymphocytes, fibroblast-like synoviocytes, and osteoclasts, although the depth of supporting evidence varies substantially across mechanisms and cellular compartments. COMMD3/8 currently provides the most complete molecular–functional evidence, integrating physical interaction with cellular and causal validation. IGF2BP3 is supported by biophysical and functional genetic evidence, whereas HDAC3 is supported by cellular target engagement, biophysical interaction, and functional and mutational evidence linking its modulation to the FOXO3–GLS1 pathway, although this mechanism has so far been demonstrated only in non-RA T-cell and colitis systems. Reproducible effects on pathogenic FLS functions and osteoclast differentiation further support activity in stromal and osteoimmune compartments. Taken together, the available studies indicate convergent, multilevel modulation of the inflammatory microenvironment. However, most proposed relationships between cell populations derive from parallel responses or multicellular systems that do not establish the direction, necessity, or persistence of intercellular signaling. Current evidence therefore does not support causal and durable reprogramming of the rheumatoid synovium.

Clinical translation remains constrained by limited human validation, poor oral exposure, potentially broad covalent protein reactivity, and a narrow therapeutic window. Translational assessment would be strengthened by linking target engagement and joint exposure to functional and structural outcomes and safety in established disease, while using appropriate controls to distinguish celastrol pharmacology from carrier-related effects. Overall, the evidence supports continued preclinical interest in celastrol but is insufficient to establish its clinical viability in RA. Its current value may lie in further mechanistic investigation and in serving as a pharmacological probe or a starting scaffold for developing more selective and safer therapeutic strategies.

## Figures and Tables

**Figure 1:**
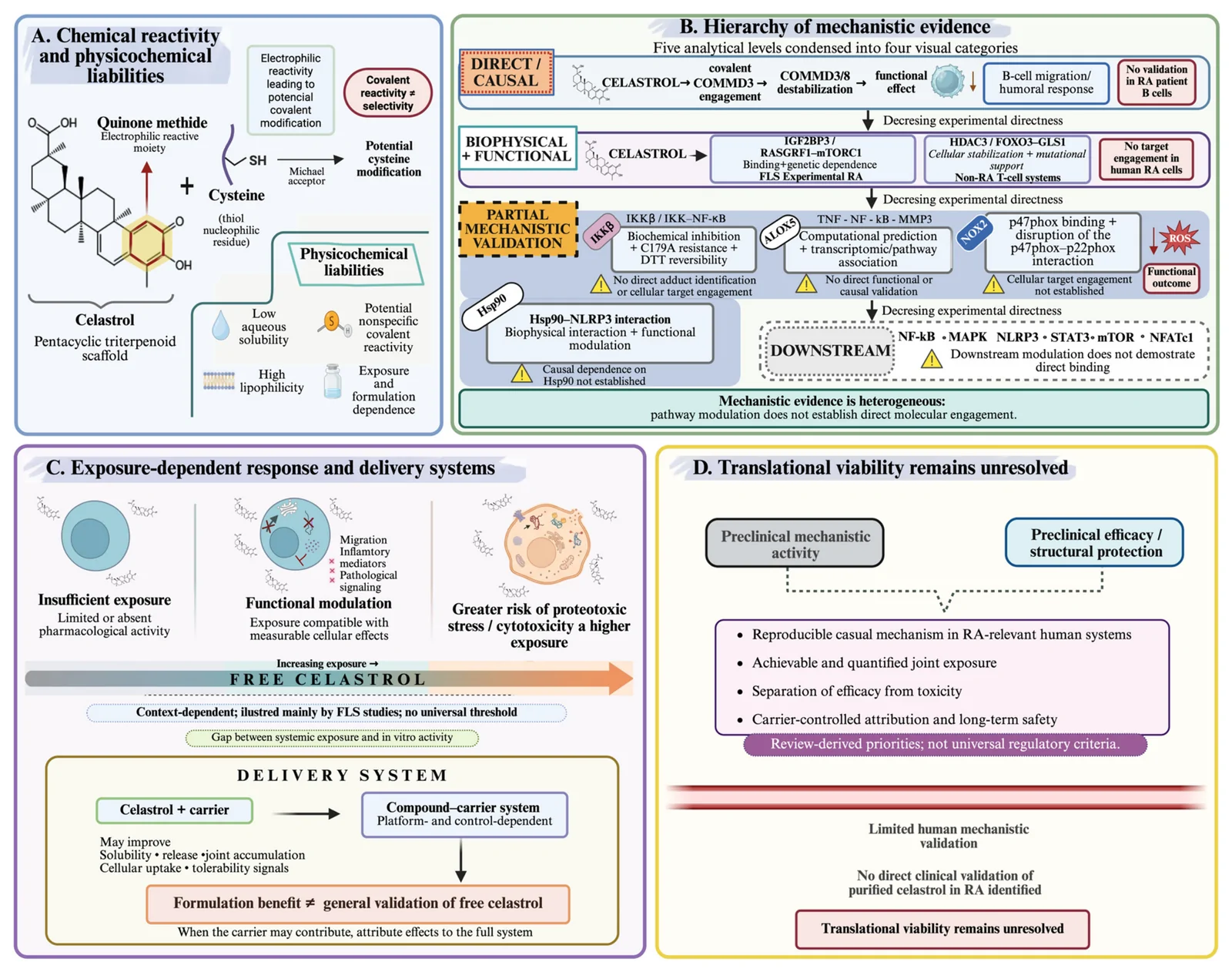
Chemical reactivity, mechanistic evidence hierarchy, and translational barriers of celastrol in rheumatoid arthritis. (**A**) Structural features underlying celastrol’s electrophilic reactivity and physicochemical limitations [15,16]. (**B**) Review-derived hierarchy organized by the directness of mechanistic evidence. COMMD3/8 has direct molecular and causal support [17]; IGF2BP3 and HDAC3 have biophysical and functional support, although the evidence for HDAC3 derives from non-RA systems [18,19]; and IKKβ has biochemical and mutational support, but no direct adduct identification or cellular target-engagement evidence [20]. ALOX5 is supported by molecular docking, transcriptomic and single-cell analyses, and pathway associations, but direct celastrol–ALOX5 binding, cellular target engagement, and functional or causal validation remain unproven [22]. NOX2 inhibition is supported by celastrol binding to recombinant p47phox, disruption of the p47phox–p22phox interaction, and functional cellular evidence [21]. The remaining signaling nodes primarily reflect downstream pathway modulation. The hierarchy is qualitative and should not be interpreted as a quantitative score, biological sequence, or measure of clinical relevance. (**C**) Exposure- and context-dependent cellular responses and the potential influence of delivery systems on celastrol activity and on attribution of effects [9,10,28,46]. When the carrier may contribute to the observed effects, the findings are attributed to the compound–carrier system. (**D**) Translational priorities derived from preclinical evidence include causal validation in RA-relevant human systems, quantification of joint exposure, separation of efficacy from toxicity, appropriate carrier controls, and long-term safety [8,44]. These priorities were developed for this review and do not constitute universal regulatory criteria. ALOX5, arachidonate 5-lipoxygenase; COMMD3/8, COMM domain-containing proteins 3 and 8; DTT, dithiothreitol; FLS, fibroblast-like synoviocyte; FOXO3, forkhead box O3; GLS1, glutaminase 1; HDAC3, histone deacetylase 3; Hsp90, heat shock protein 90; IGF2BP3, insulin-like growth factor 2 mRNA-binding protein 3; IKK, IκB kinase; IKKβ, IκB kinase beta; MAPK, mitogen-activated protein kinase; MMP-3, matrix metalloproteinase-3; mTOR, mechanistic target of rapamycin; mTORC1, mechanistic target of rapamycin complex 1; NFATc1, nuclear factor of activated T cells 1; NF-κB, nuclear factor kappa B; NLRP3, NLR family pyrin domain-containing 3; NOX2, NADPH oxidase 2; p22phox, 22 kDa phagocyte oxidase subunit; p47phox, 47 kDa phagocyte oxidase subunit; RA, rheumatoid arthritis; RASGRF1, Ras protein-specific guanine nucleotide-releasing factor 1; ROS, reactive oxygen species; STAT3, signal transducer and activator of transcription 3; TNF, tumor necrosis factor. Created in BioRender. Hernández Ortega, L. D. (2026) https://BioRender.com/ul2umqp (accessed on 18 September 2026).

**Figure 2:**
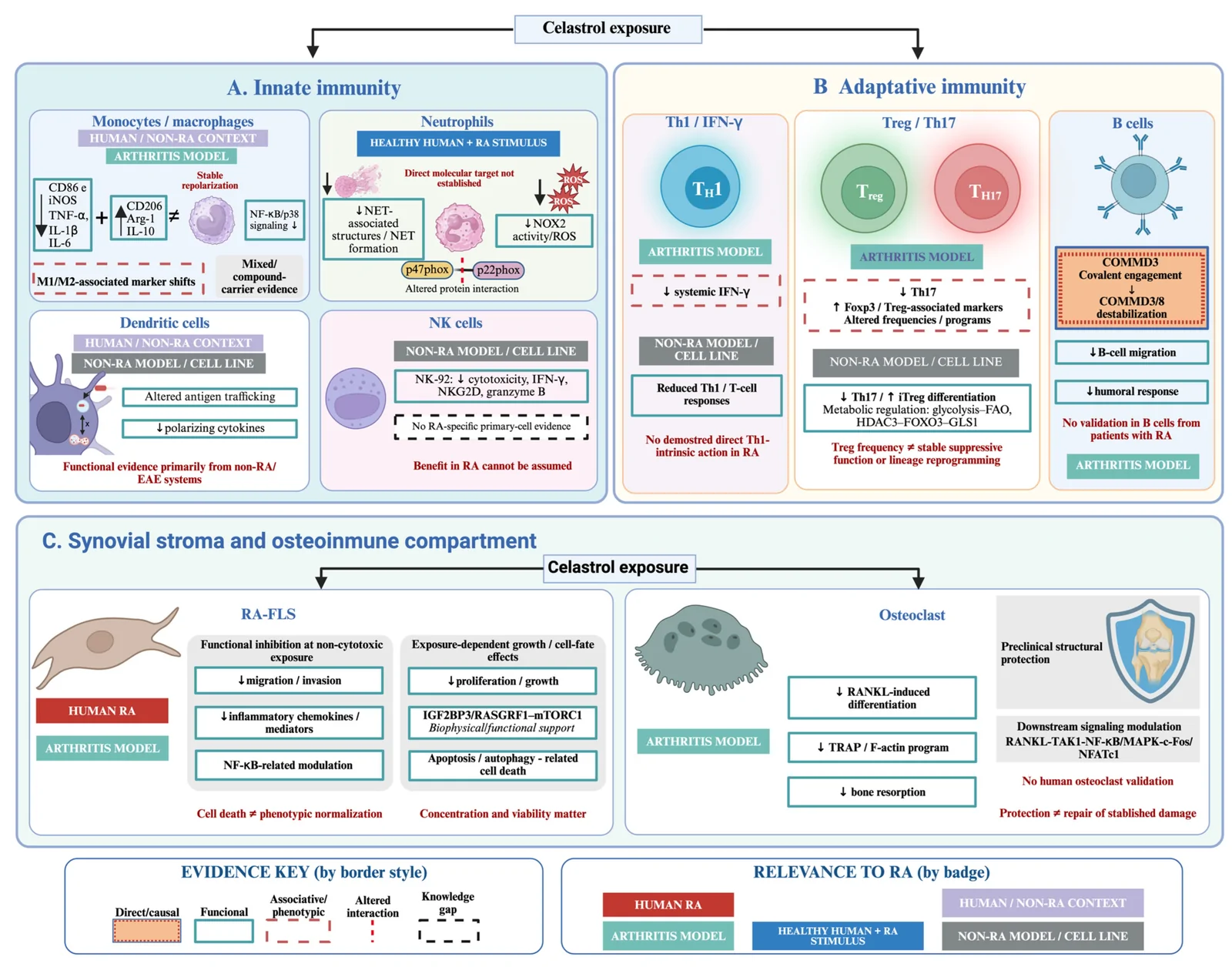
Cellular evidence landscape of celastrol in rheumatoid arthritis. (**A**) Innate immune evidence includes effects on monocytes/macrophages, neutrophils, dendritic cells, and natural killer cells across arthritis models, healthy human cells exposed to RA-related stimuli, and non-RA experimental systems. Macrophage findings are consistent with shifts in M1/M2-associated markers rather than stable repolarization [32,43,45,47,50]. In neutrophils, celastrol reduces ROS- and NET-associated responses and inhibits NOX2 through p47phox binding and disruption of the p47phox–p22phox interaction [21,33,56]. Evidence involving dendritic and natural killer cells is predominantly derived from non-RA systems and therefore remains extrapolative for RA [57,58,63]. (**B**) Adaptive immune evidence includes modulation of Th1/IFN-γ responses [57,59,64], the Treg/Th17 balance [36,38,58,67], and B-cell function [17,69]. Changes in Treg-associated markers or frequency do not establish stable suppressive function or lineage reprogramming. The HDAC3–FOXO3–GLS1 axis is supported by mechanistic evidence in non-RA T-cell systems [19], whereas COMMD3/8 provides the strongest direct molecular–functional evidence in B cells, although validation in B cells from patients with RA is lacking [17]. (**C**) In the synovial stromal and osteoimmune compartments, celastrol inhibits migration, invasion, inflammatory mediator production, and other functional responses in RA-FLS [27,29,31,40], while higher or context-dependent exposures can reduce viability and induce autophagy-dependent cytotoxicity or apoptosis [27,40,41]. IGF2BP3/RASGRF1–mTORC1 signaling has biophysical and functional support in RA-relevant FLS systems [18]. Celastrol also suppresses RANKL-induced osteoclast differentiation, the TRAP/F-actin program, and bone resorption, with downstream modulation of TAK1, NF-κB/MAPK, c-Fos, and NFATc1 [34,39,44]. These findings support preclinical structural protection but do not demonstrate repair of established damage [34,39,44]. Border styles indicate the directness of the experimental evidence, whereas badges indicate the relevance of the experimental system to RA. Findings involving active carriers are identified as mixed compound–carrier evidence and should not be attributed to free celastrol alone. Convergent cellular effects do not establish causal intercellular communication or persistent reprogramming. Arg-1, arginase 1; COMMD3, COMM domain-containing protein 3; EAE, experimental autoimmune encephalomyelitis; FAO, fatty acid oxidation; F-actin, filamentous actin; Foxp3, forkhead box P3; IFN-γ, interferon gamma; IL-1β, interleukin-1 beta; IL-6, interleukin-6; IL-10, interleukin-10; iNOS, inducible nitric oxide synthase; iTreg, induced regulatory T cell; M1, M1-like macrophage phenotype; M2, M2-like macrophage phenotype; NET, neutrophil extracellular trap; NK, natural killer; NKG2D, natural killer group 2 member D; p38, p38 mitogen-activated protein kinase; RA-FLS, rheumatoid arthritis fibroblast-like synoviocytes; RANKL, receptor activator of nuclear factor kappa B ligand; TAK1, transforming growth factor beta-activated kinase 1; Th1, T helper 1 cell; TNF-α, tumor necrosis factor alpha; TRAP, tartrate-resistant acid phosphatase; Treg, regulatory T cell. Created in BioRender. Hernández Ortega, L. D. (2026) https://BioRender.com/0b1abd7 (accessed on 18 September 2026).

**Figure 3:**
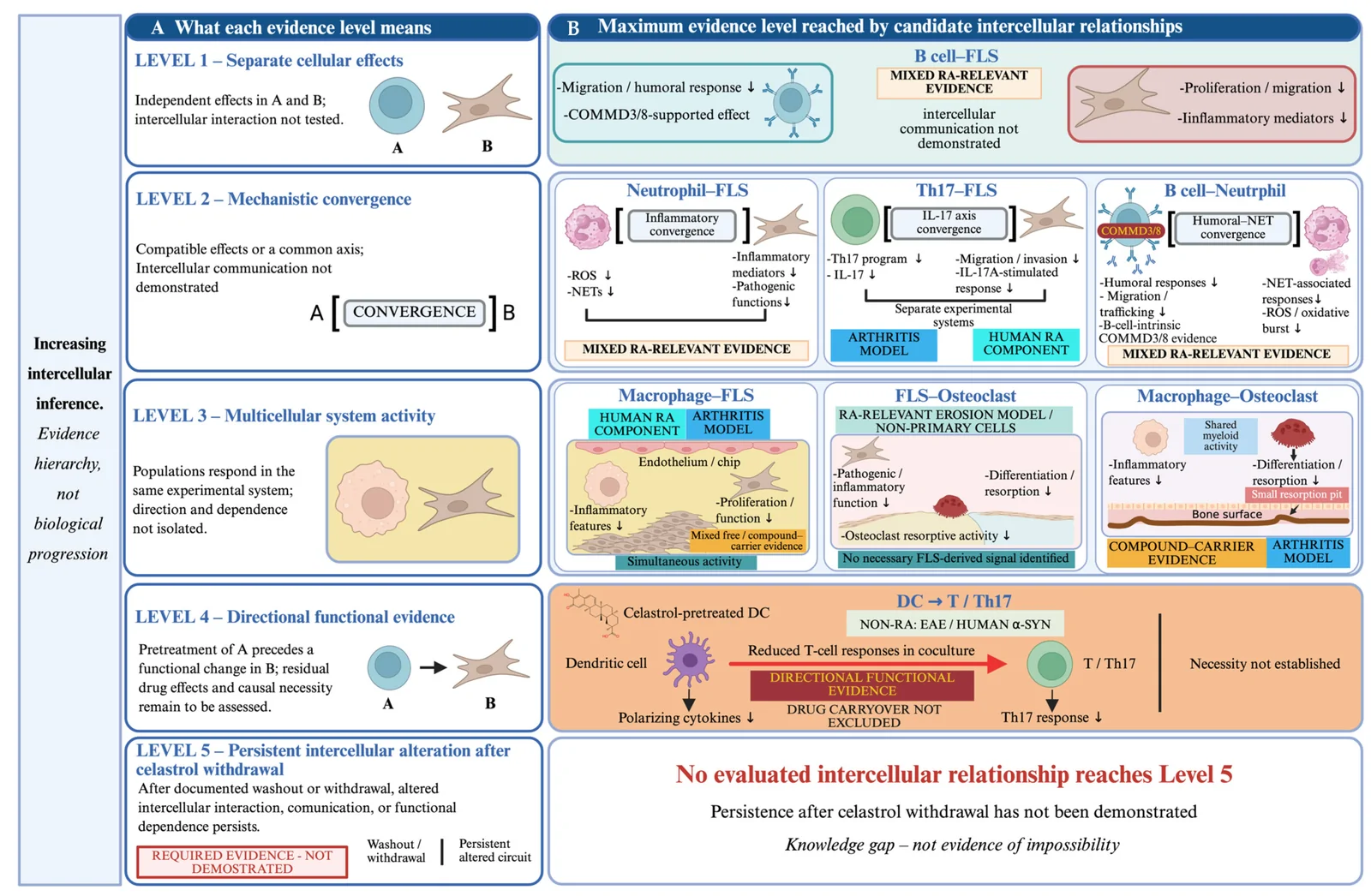
Hierarchy of intercellular evidence for celastrol in rheumatoid arthritis. (**A**) Intercellular evidence was classified by the maximum inference supported by the experimental design: Level 1, separate cellular effects; Level 2, mechanistic convergence without demonstrated intercellular transmission; Level 3, activity within a multicellular system without isolated direction or dependence; Level 4, directional functional evidence from experiments evaluating the influence of a celastrol-conditioned cell population on another; Level 5, persistent alteration after celastrol withdrawal. The hierarchy reflects the strength of intercellular inference rather than biological progression, therapeutic importance, or RA relevance. (**B**) Current evidence places B cell–FLS at Level 1 [17,29]; neutrophil–FLS [29,33], Th17–FLS [31,36], and B cell–neutrophil [17,33] at Level 2; macrophage–FLS [11,12], FLS–osteoclast [34,77], and macrophage–osteoclast [48] at Level 3; and DC–T/Th17 at Level 4 [57,58]. For DC–T/Th17, potential residual drug exposure and the lack of DC-specific necessity or rescue experiments limit causal attribution. Level 4 evidence derives from non-RA systems, and no evaluated relationship attains Level 5. Findings involving active delivery systems are attributed to the compound–carrier system where appropriate [12,48]. COMMD3/8, COMM domain-containing proteins 3 and 8; DC, dendritic cell; EAE, experimental autoimmune encephalomyelitis; FLS, fibroblast-like synoviocyte; IL-17, interleukin-17; IL-17A, interleukin-17A; NET, neutrophil extracellular trap; RA, rheumatoid arthritis; ROS, reactive oxygen species; Th17, T helper 17 cell. Created in BioRender. Hernández Ortega, L. D. (2026). https://BioRender.com/6aqfuu1 (accessed on 18 September 2026).

**Table 1:** Molecular evidence hierarchy for candidate targets and celastrol mechanisms relevant to rheumatoid arthritis.

Target/Mechanism	Evidence of Physical Interaction	Relevant Cell/Model	Functional Evidence/Causal Validation	Relevance to RA	Maximum Category of Evidence Reached	Main Limitation	Reference(s)
**COMMD3/8**	Covalent Binding to COMMD3 and MS Identification of a Modified Cysteine Residue	B lymphocytes; Murine model of autoimmunity/arthritis	Disruption of the COMMD3/8 complex; inhibition of B-cell migration; reduced humoral responses and arthritis severity; a resistant COMMD3 variant abolished complex disruption and the therapeutic effect	Preclinical evidence directly relevant to inflammatory arthritis	Physical interaction + cellular target engagement + functional/causal validation	Single study without independent replication or validation in B cells from patients with RA; the findings do not establish that COMMD3/8 accounts for all celastrol-associated activity	[17]
**IGF2BP3/RASGRF1–mTORC1 axis**	Biophysical binding demonstrated by SPR (KD = 1.65 µM); complementary Bio-CEL colocalization and pull-down assays using RA-FLS and RAW264.7 cell lysates, with additional evidence from RA synovial tissue lysates	FLS and other relevant cell systems; experimental arthritis in IGF2BP3-deficient animals	Inhibition of cellular proliferation and activation was associated with modulation of the IGF2BP3–RASGRF1–mTORC1 axis; the anti-arthritic effect of celastrol depended on IGF2BP3 in the genetic model	Preclinical evidence directly relevant to RA	Biophysical interaction + functional/genetic validation	Single study without independent replication; although Bio-CEL assays provide complementary interaction evidence, definitive target engagement by unmodified celastrol in intact human RA cells remains to be established	[18]
**HDAC3/FOXO3–GLS1 axis**	MST using HEK293T lysates expressing human HDAC3 demonstrated celastrol binding (WT KD = 42.1 nM), whereas G143A showed no detectable binding under the reported assay conditions. Thermal stabilization of HDAC3 was also observed in celastrol-treated Th17 cells	Murine CD4^+^ T cells under Th17-polarizing conditions; DSS-induced colitis	HDAC3 inhibition increased FOXO3-dependent repression of GLS1 and reduced glutaminolysis and Th17 differentiation; GLS1 overexpression, FOXO3 silencing, HDAC3 overexpression, and G143A mutagenesis attenuated celastrol-associated responses	Mechanistically relevant to Th17 regulation, but validated in non-RA systems	Biophysical interaction + functional/mutational validation; supportive cell-associated thermal stabilization	Single study without independent replication; lysate-based binding and thermal-shift assays do not definitively establish target engagement in intact cells. The mechanism remains unvalidated in RA models or T cells from patients with RA	[19]
**IKK** **β** **/IKK–NF-** **κ** **B**	Biochemical inhibition consistent with cysteine-dependent interaction; C179A mutation confers resistance; dithiothreitol reverses inhibition	Transfected and inflammatory cellular systems; non-RA in vivo models	Celastrol inhibited IKKβ activity and downstream NF-κB signaling; substitution of Cys179 reduced sensitivity to celastrol	Indirect RA relevance	Biochemical evidence consistent with interaction + functional/mutational validation	No direct identification of a celastrol–IKKβ adduct or cellular target-engagement assay; evidence was obtained outside RA-specific human systems	[20]
**ALOX5: predicted binding and associated TNF–NF-κB/metalloproteinase pathways**	No experimentally demonstrated physical interaction; molecular docking predicted a potential celastrol–ALOX5 binding mode	RA PBMC, whole-blood, and single-cell transcriptomic datasets; macrophage-enriched ALOX5 expression; public genetic-perturbation datasets analyzed in silico	No direct functional or causal validation of the celastrol–ALOX5 relationship. Public genetic-perturbation data associated ALOX5 knockout with reduced MMP2 expression and, separately, knockout of shared transcription factors with reduced TNF–NF-κB pathway activity. Observational analyses associated ALOX5 expression with MMP3 protein levels	RA-relevant computational, transcriptomic, single-cell, and pathway-level evidence	Computational prediction + transcriptomic/pathway association	No direct binding, cellular target engagement, enzymatic assay, or experimental demonstration that ALOX5 is required for celastrol activity; all mechanistic conclusions were derived from in silico analyses	[22]
**Hsp90–NLRP3 interaction**	Molecular docking predicted a celastrol–Hsp90 binding mode, and Octet RED96 measurements indicated an interaction; however, the reported Kd (1.04 mM) is inconsistent with the nanomolar celastrol concentrations specified for the binding assay	TNF-α-stimulated FLS and a murine CIA model	Celastrol reduced NLRP3 coimmunoprecipitation with Hsp90 and decreased NLRP3, ASC, and cleaved caspase-1; Hsp90-specific causal dependence was not established	RA-relevant preclinical evidence	Reported biophysical interaction, with unresolved quantitative affinity, and associative protein-interaction changes	The reported binding affinity is discordant with the assay concentrations. Reduced total NLRP3 may contribute to the lower coimmunoprecipitation signal. No target-specific perturbation or rescue established that Hsp90 binding is required for celastrol responsiveness	[26]
**NOX2 inhibition through p47phox binding and disruption of the p47phox–p22phox interaction**	Direct binding of celastrol to recombinant p47phox was supported by intrinsic fluorescence and native mass spectrometry; a pull-down assay showed disruption of the p47phox–p22phox interaction	Cell-free and recombinant protein systems; human NOX-expressing cellular systems and neutrophils from healthy donors	Celastrol inhibited NOX2-dependent ROS production and disrupted the p47phox–p22phox interaction	Mechanistically relevant to RA-associated neutrophil responses, but validated in non-RA experimental systems	Biophysical interaction + biochemical mechanism + cellular functional validation	The binding site and chemical nature of the celastrol–p47phox interaction were not established; no cellular target-engagement, mutational, or rescue experiment demonstrated dependence on p47phox, and the mechanism was not validated in neutrophils from patients with RA or in an arthritis model	[21]
**Selected downstream nodes: NF-κB, MAPK, NLRP3, STAT3, mTOR, NFATc1, and Nrf2/HO-1/GPX4**	Direct celastrol binding to these nodes has not been demonstrated in the evidence evaluated	Macrophages, neutrophils, T cells, RA-FLS, and osteoclast-related systems; experimental arthritis and selected non-RA inflammatory models	Celastrol altered the phosphorylation, expression, localization, or activity of these signaling nodes, together with corresponding changes in inflammatory, metabolic, survival, and osteoclastogenic responses	Variable, ranging from directly RA-relevant preclinical evidence to evidence from non-RA inflammatory systems	Downstream pathway modulation, with functional perturbation support for selected pathways	Downstream modulation and pathway perturbation do not establish direct molecular binding or demonstrate that each node is required for the integrated anti-arthritic effect	NF-κB/MAPK: [26,27,28,29,30,31,32,33,34]; NLRP3: [26,30,35]; STAT3: [36]; mTOR: [18,27,37,38]; NFATc1: [34,39]; Nrf2/HO-1/GPX4: [40].

**Note.** The evidence categories were developed for this narrative review to summarize the depth and directness of mechanistic support and should not be interpreted as a validated quantitative score or a measure of clinical relevance. RA relevance was considered independently of mechanistic depth. ALOX5, arachidonate 5-lipoxygenase; ASC, apoptosis-associated speck-like protein containing a caspase recruitment domain; CD4, cluster of differentiation 4; CIA, collagen-induced arthritis; COMMD3, COMM domain-containing protein 3; COMMD3/8, COMM domain-containing proteins 3 and 8; DSS, dextran sulfate sodium; FLS, fibroblast-like synoviocyte; FOXO3, forkhead box O3; GLS1, glutaminase 1; GPX4, glutathione peroxidase 4; HDAC3, histone deacetylase 3; HO-1, heme oxygenase-1; Hsp90, heat shock protein 90; IGF2BP3, insulin-like growth factor 2 mRNA-binding protein 3; IKK, IκB kinase; IKKβ, IκB kinase beta; MAPK, mitogen-activated protein kinase; MMP-2, matrix metalloproteinase-2; MMP-3, matrix metalloproteinase-3; MS, mass spectrometry; mTOR, mechanistic target of rapamycin; mTORC1, mechanistic target of rapamycin complex 1; NFATc1, nuclear factor of activated T cells 1; NF-κB, nuclear factor kappa B; NLRP3, NLR family pyrin domain-containing 3; NOX2, NADPH oxidase 2; Nrf2, nuclear factor erythroid 2-related factor 2; p22phox, 22 kDa phagocyte oxidase subunit; p47phox, 47 kDa phagocyte oxidase subunit; RA, rheumatoid arthritis; RA-FLS, rheumatoid arthritis fibroblast-like synoviocyte; RASGRF1, Ras protein-specific guanine nucleotide-releasing factor 1; ROS, reactive oxygen species; SPR, surface plasmon resonance; STAT3, signal transducer and activator of transcription 3; Th17, T helper 17 cell; TNF, tumor necrosis factor; TNF-α, tumor necrosis factor alpha.

**Table 2:** Experimental evidence of celastrol in immune, stromal, and osteoimmune compartments relevant to rheumatoid arthritis.

Cell Compartment	Experimental System/Relevance for RA	Celastrol Free or Formulation	Main Functional Effects	Main Mechanistic Evidence	Directness of the Evidence	Critical Limitation	Reference(s)
**Monocytes/macrophages**	AIA and CIA; RAW264.7 macrophages; monocyte-derived human macrophages; formulation-based macrophage models	Both; core data include free celastrol and targeted or delivery-enhancing formulations, with selected direct comparisons between free celastrol and formulated celastrol	↓ synovial macrophages and inflammatory mediators; ↓ NF-κB/p38 and lipid mediators; shifts in M1/M2-associated markers; formulation-enhanced uptake and cytokine modulation in selected models	NF-κB is the most consistently reproduced functional node; p38 and lipid metabolism are also modulated; M1/M2-associated marker changes do not establish stable repolarization; formulation studies provide receptor-associated uptake or downstream cytokine/pathway evidence	Preclinical evidence directly relevant to RA + functional support in human cells, with variable mechanistic and formulation attribution by system	Limited validation in primary RA synovial macrophages; reliance on murine models and formulations; M1/M2-associated changes do not establish stable conversion; cytotoxicity may contribute, and formulation benefits are rarely supported by quantitative celastrol exposure data	[28,32,43,45,47,50]
**Neutrophils**	Healthy human neutrophils stimulated with TNF-α, immune complexes, or IgG from patients with RA; cell-free system; nanocarrier-based CIA model	Predominantly free; also neutrophil-targeted formulation	↓ ROS and oxidative burst; ↓ release of NET-associated structures and histone H3 citrullination; targeted formulation also induced apoptosis	Biochemical inhibition of NOX2 and altered p47phox–p22phox interaction; ↓ SYK–MEK–ERK–NF-κB downstream signaling	RA-relevant human functional evidence, but in healthy donor cells; indirect in vivo evidence	No studies treated neutrophils isolated from patients with RA; absence of neutrophil-specific causality in vivo; NET-associated assays have methodological limitations; formulation lacks equivalent comparison with free celastrol	[21,33,56]
**Dendritic cells (DC)**	Healthy-donor monocyte-derived DCs in a human α-synuclein-specific T-cell coculture model; murine BMDC–T-cell cocultures and splenic DCs from celastrol-treated EAE mice (non-RA)	Free in major mechanistic studies	Altered antigen trafficking and cytokine production; celastrol-conditioned DCs reduced Th1 and Th17 responses in non-RA cocultures, alongside reduced Treg frequency in the human model; systemic celastrol also attenuated adoptive-transfer EAE following lymphocyte transfer	Endosomal/autophagic trafficking and IKK–NF-κB signaling; functional DC→T-cell evidence in EAE	Directional functional evidence from non-RA cocultures; DC-specific causal necessity remains unestablished	There is no equivalent demonstration with DC of patients with RA, arthrogenic autoantigens, or joint models; altered antigen trafficking and reduced T-cell responses do not establish stable tolerance	[57,58]
**NK cells**	NK-92 cell line in a non-RA system	Free	↓ cytotoxicity, IFN-γ production, NKG2D expression, and granzyme B	Pharmacological inhibition of c-Myb; c-Myb silencing reproduced part of the phenotype	Direct cellular evidence, but extrapolative and non-RA	No data are available from primary circulating or synovial NK cells in RA; the multitarget nature of celastrol precludes exclusive attribution to c-Myb	[63]
**Th1/IFN-γ**	CIA with systemic measurement of IFN-γ; t cells and DC–T systems in lupus/α-synuclein outside RA	Free	↓ systemic IFN-γ in arthritis models; ↓ Th1- or T-cell–specific responses in non-RA models	In experimental arthritis, evidence is limited to systemic cytokine changes; non-RA studies report cellular T-cell responses and DC-associated changes in antigen processing	Indirect RA evidence + extrapolative cellular evidence	Serum IFN-γ does not identify its source; there is no controlled Th1 polarization with CD4+ of RA, T-bet/STAT4, viability and rescue demonstrating T-intrinsic action	[57,59,64]
**Treg/Th17**	Arthritis models; EAE with DC–T co-culture; AIA with nanoplatform	Both	Reduced Th17-associated responses and increased Treg-associated markers in arthritis models; celastrol-conditioned DCs reduced Th17 responses in non-RA cocultures; a celastrol-loaded nanoplatform increased Foxp3^+^CD25^+^ cell frequency	STAT3-associated signaling in AIA and mTOR/AMPK-associated metabolic changes in murine T-cell polarization experiments; HDAC3–FOXO3–GLS1 supported by biophysical and functional/mutational evidence in non-RA systems; NF-κB-associated modulation in murine BMDCs	RA-relevant preclinical phenotypic and pathway evidence; non-RA functional/mutational validation for HDAC3 and directional DC–T coculture evidence without established DC-specific causal necessity	Foxp3/Treg frequency does not demonstrate suppressive function or lineage stability; epigenetic stability and post-withdrawal persistence were not assessed; carrier-related effects remain possible; HDAC3–FOXO3–GLS1 has not been validated in RA T cells or arthritis models	[19,36,38,58,67]
**B lymphocytes**	Murine models of humoral response and arthritis/autoimmunity	Free	↓ B-cell migration and humoral responses; reduced arthritis severity; ↓ autoantibody levels in additional models	Covalent binding to COMMD3, destabilization of COMMD3/8 and loss of effect with resistant variant	Strong preclinical causal molecular and functional evidence	No validation in B cells from RA patients; no direct action on proliferation, plasma differentiation, memory, regulatory B cells, affinity, or tolerance; COMMD3/8 does not necessarily explain the entire humoral response	[17,69]
**RA-FLS**	Primary FLS from patients with RA; FLS from arthritic joints; TNF-α-stimulated rat FLS; experimental arthritis models	Predominantly free celastrol in the main mechanistic evidence	↓ proliferation, migration, invasion, chemokines, and inflammatory mediators; apoptosis/cell-cycle arrest, autophagy-dependent cell death, and ferroptosis-associated responses in selected settings	NF-κB as a downstream signaling node; Ca^2+^/CaMKKβ/AMPK–mTOR signaling with functional rescue; IGF2BP3/RASGRF1–mTORC1 signaling with biophysical and genetic support; PTGS2-associated ferroptosis resistance with functional perturbation; Nrf2/HO-1/GPX4 pathway involvement supported by pharmacological perturbation	Human cellular evidence directly relevant to RA, with some functionally validated mechanisms	Few donors, poor characterization of FLS heterogeneity, and potential overlap between pathway modulation and cytotoxicity; stable reprogramming after celastrol withdrawal has not been demonstrated; Nrf2/HO-1/GPX4 involvement does not establish direct molecular target engagement	[18,27,29,31,37,40,41,75]
**Osteoclasts**	BMMs/RAW264.7 cells stimulated with M-CSF/RANKL; CIA, AIA, and other bone-loss models	Predominantly free in central mechanistic and structural studies	↓ TRAP^+^ cells, F-actin rings, osteoclastogenic program, and bone resorption; ↓ preclinical bone erosion with structural protection	RANKL–TAK1–NF-κB/MAPK–c-Fos/NFATc1 signaling; coexistence of direct effects on osteoclast precursors and indirect attenuation of osteoclastogenic signals	Preclinical direct functional evidence + relevant structural outcomes for RA	No studies in celastrol-treated human osteoclasts; incomplete distinction between inhibition of osteoclastogenesis and cytotoxic depletion; direct versus indirect effects not quantified; possible inhibition of osteoblast function; structural protection does not demonstrate repair or post-treatment persistence	[6,34,39,44,78]

**Note.** The classification of evidence integrates the relevance of the experimental system to RA and the depth of functional validation and should not be interpreted as a formal quality score. Changes in cellular markers or signaling pathways do not by themselves demonstrate stable cellular reprogramming or identify a direct molecular target. When the carrier may contribute to the observed effects, findings are attributed to the compound–carrier system.

**Table 3:** Experimental evidence distinguishing cellular convergence from causal crosstalk during celastrol treatment.

Proposed Cell Circuit	Experimental System	Celastrol Intervention	Multicellular/Convergent Observation	Intercellular Interaction Directly Tested?	Causal Perturbation/Rescue	Maximum Level Reached	Main Interpretation and Limitation	Reference(s)
**Macrophage–FLS/myeloid–stromal interface**	Synovium-on-a-chip containing RA-FLS, M1 macrophages, and endothelial cells; CIA treated with an HA-based celastrol nanomedicine hydrogel	Free celastrol in the synovium-on-a-chip model; celastrol-containing compound–carrier system in the hydrogel model	Celastrol showed activity in a multicellular synovial environment; the hydrogel system reduced macrophage proinflammatory features and FLS proliferation	Partially. Conditioned-medium experiments assessed macrophage- and RA-FLS-related responses, but did not establish causal intercellular dependence or fully exclude residual celastrol or carrier effects	No. No mediator neutralization, ligand–receptor blockade, cell-specific perturbation, or rescue experiment demonstrated macrophage-to-FLS or FLS-to-macrophage dependence	Level 3: activity in a multicellular system	These models provide directly RA-relevant evidence of multicellular activity, including conditioned-medium responses, but do not establish mediator-specific causal dependence between macrophages and FLS. Effects observed with the hydrogel are attributed to the compound–carrier system	[11,12]
**Neutrophil–FLS/neutrophil–stroma**	Neutrophils from healthy donors stimulated with RA IgG, TNF-α, or immune complexes; RA-FLS evaluated in separate experimental systems	Free celastrol in separate neutrophil and RA-FLS experiments	Celastrol reduced ROS- and NET-associated responses in neutrophils and, in separate RA-FLS systems, reduced NF-κB signaling, chemokine production, migration, and invasion	No. Neutrophils and RA-FLS were evaluated in separate experimental systems, without direct or indirect coculture	No. Neutrophil-conditioned medium or isolated NET-associated material was not applied to RA-FLS, and no mediator blockade or rescue experiment tested neutrophil-to-FLS dependence	Level 2: mechanistic convergence	Parallel effects in neutrophils and RA-FLS support multicellular convergence but do not demonstrate that celastrol-induced changes in neutrophils modify the RA-FLS phenotype	[29,31,33]
**DC–T/DC–Th17**	Murine BMDC-naïve T-cell cocultures and splenic DCs isolated from celastrol-treated EAE mice; human monocyte-derived DCs and α-synuclein-specific T-cell cocultures using healthy-donor cells	Free celastrol; in vitro DC pretreatment and ex vivo isolation of DCs from celastrol-treated EAE mice	Celastrol-conditioned DCs reduced murine Th17 responses and human antigen-specific Th1 and Th17 responses, alongside reduced Treg frequency in the human model	Yes. DC pretreatment followed by T-cell coculture provided directional functional evidence, although residual celastrol exposure was not conclusively excluded	No DC-specific necessity or rescue experiments. Adoptive transfer involved lymphocytes rather than treated DCs	Level 4: directional functional evidence in non-RA systems, with unresolved drug carryover and causal necessity	DC-conditioned modulation of T-cell responses supports directional functional evidence. However, definitive DC-specific causal necessity, RA-specific validation, and persistence after celastrol withdrawal remain unestablished	[57,58]
**Th17–FLS**	AIA for Th17/Treg-associated responses; IL-17A-stimulated human RA-FLS evaluated in a separate experimental system	Free celastrol in separate AIA and RA-FLS experiments	Celastrol reduced Th17-associated responses and increased Foxp3-associated measures in AIA and, separately, reduced migration and invasion of IL-17A-stimulated RA-FLS	No. Th17-associated responses and IL-17A-responsive RA-FLS were evaluated in separate experimental systems	No. No Th17–FLS coculture, Th17-conditioned medium, IL-17/IL-17R blockade, or rescue experiment tested Th17-to-FLS dependence	Level 2: convergence on the IL-17 axis	Parallel modulation of Th17-associated responses and IL-17A-responsive RA-FLS supports convergence on the IL-17 axis but does not demonstrate celastrol-dependent alteration of Th17-to-FLS signaling	[31,36]
**B–neutrophil/humoral response–NET**	Murine models of humoral immunity and autoimmunity; neutrophils from healthy donors stimulated with RA IgG or immune complexes in a separate experimental system	Free celastrol in separate B-cell/humoral and neutrophil experiments	Celastrol reduced B-cell migration and humoral responses through COMMD3/8 disruption and, separately, reduced IgG- or immune complex-induced ROS- and NET-associated responses in neutrophils	No. Humoral and neutrophil responses were evaluated in separate experimental systems	No. Antibodies generated during celastrol treatment were not isolated and tested for their ability to activate neutrophils, and no antibody-transfer or rescue experiment evaluated B-cell-to-neutrophil dependence	Level 2: multilevel convergence	Causal COMMD3/8-dependent modulation within B cells and direct inhibition of IgG-responsive neutrophil functions support multilevel convergence but do not establish a B-cell-to-antibody-to-neutrophil causal sequence	[17,33]
**B–FLS**	B lymphocytes and RA-FLS evaluated in separate experimental systems	Free celastrol in separate B-cell and RA-FLS experiments	Celastrol inhibited COMMD3/8-dependent B-cell migration and, separately, reduced inflammatory and pathogenic functions of RA-FLS	No. B lymphocytes and RA-FLS were evaluated in separate experimental systems	No. No B-cell–FLS coculture, adhesion assay, conditioned-medium experiment, mediator blockade, or rescue experiment tested intercellular dependence	Level 1: separate cellular effects	Celastrol affects B-cell and RA-FLS functions in separate systems, but no evidence demonstrates celastrol-dependent modulation of B-cell–FLS communication	[17,29]
**FLS–osteoclast/stroma–erosion module**	Microfluidic chip containing SW982 cells (non-primary human synovial cell line), RANKL-stimulated RAW264.7-derived osteoclasts, and osteogenically induced bone marrow mesenchymal stem cells; complementary RANKL-based osteoclast systems.	Free celastrol in the microfluidic and complementary osteoclast experiments	Celastrol reduced SW982 cell migration and osteoclast-associated TRAP activity within the same microfluidic system; direct inhibition of osteoclast differentiation and bone resorption was demonstrated in complementary RANKL-based models	Partially. Stromal, osteoclastic, and osteogenic cells were integrated into the same microfluidic system, but directional intercellular dependence was not isolated	No. No conditioned-medium experiment, cell-specific perturbation, RANKL/OPG blockade, or rescue experiment demonstrated that the osteoclast response depended on celastrol-induced changes in the stromal compartment	Level 3: activity within a multicellular system	The integrated system demonstrates concurrent stromal and osteoclast-associated responses to celastrol but does not establish a necessary FLS-to-osteoclast signaling mechanism	[34,77]
**Macrophage–osteoclast/inflammation–resorption**	Advanced AIA model; complementary uptake and apoptosis experiments using synovial macrophages and peripheral blood-derived osteoclasts from patients with advanced RA.	MMP-9-responsive, RGD-modified celastrol-loaded nanoparticles	The nanoparticle system promoted apoptosis of inflammatory macrophages and osteoclasts and was associated with reduced inflammation and bone erosion	No. Both populations were targeted within the same intervention, but signaling or functional dependence between macrophages and osteoclasts was not tested	No. Both populations were directly targeted, and no cell-specific perturbation, depletion, transfer, or rescue experiment demonstrated macrophage-to-osteoclast or osteoclast-to-macrophage dependence	Level 3: multicellular targeting and activity	Simultaneous targeting of macrophages and osteoclasts was associated with inflammatory and structural benefit but did not establish causal intercellular dependence. The findings are attributed to the compound–carrier system	[48]

**Note**. Evidence levels indicate the maximum strength of inference regarding intercellular communication: Level 1, separate cellular effects; Level 2, mechanistic convergence without demonstrated intercellular transmission; Level 3, activity within a multicellular system without isolated direction or dependence; Level 4, directional functional evidence from experiments evaluating the influence of a celastrol-conditioned cell population on another; Level 5, persistent alteration after celastrol withdrawal. The levels do not indicate biological progression, RA relevance, or clinical validity. Evidence from non-RA models is considered extrapolative, and no evaluated circuit reaches Level 5.

## Data Availability

No new data were created or analyzed in this study. Data sharing is not applicable to this article.

## Abbreviations

The following abbreviations are used in this manuscript:

ACPA	anti-citrullinated protein antibodies
AIA	Adjuvant-induced arthritis
AKT	Protein kinase B
ALOX5	Arachidonate 5-lipoxygenase
AMPK	AMP-activated protein kinase
Arg-1	Arginase 1
ARRIVE	Animal Research: Reporting of In Vivo Experiments
ASC	Apoptosis-associated speck-like protein containing a caspase activation and recruitment domain
AUC	Area under the concentration–time curve
BAFF	B-cell activating factor
BAFF-R	B-cell activating factor receptor
Bax	BCL2-associated X protein
Bcl-2	B-cell lymphoma 2
BMM	Bone marrow-derived macrophage
CaMKKβ	Calcium/calmodulin-dependent protein kinase kinase beta
CCL2	C-C motif chemokine ligand 2
CCR2	C-C motif chemokine receptor 2
CD4	Cluster of differentiation 4
CD25	Cluster of differentiation 25
CD68	Cluster of differentiation 68
CD86	Cluster of differentiation 86
CD206	Cluster of differentiation 206
CIA	Collagen-induced arthritis
Cmax	Maximum plasma concentration
COMMD3	COMM domain-containing protein 3
COMMD3/8	COMM domain-containing proteins 3 and 8
CXCL10	C-X-C motif chemokine ligand 10
CXCL12	C-X-C motif chemokine ligand 12
CXCR4	C-X-C chemokine receptor type 4
DC	Dendritic cell
DSS	Dextran sulfate sodium
DTT	Dithiothreitol
EAE	Experimental autoimmune encephalomyelitis
ERK	Extracellular signal-regulated kinase
F-actin	filamentous actin
FAO	Fatty acid oxidation
FLS	Fibroblast-like synoviocyte
FOXO3	Forkhead box O3
Foxp3	Forkhead box P3
Gαq	G protein alpha q subunit
GLS1	Glutaminase 1
GPX4	Glutathione peroxidase 4
HDAC3	Histone deacetylase 3
HIF-1α	Hypoxia-inducible factor 1 alpha
HO-1	Heme oxygenase-1
Hsp90	Heat shock protein 90
IFN-γ	Interferon gamma
IGF2BP3	Insulin-like growth factor 2 mRNA-binding protein 3
IgG	Immunoglobulin G
IκBα	inhibitor of nuclear factor kappa B alpha
IKK	IκB kinase
IKKβ	IκB kinase beta
IL-1β	Interleukin-1 beta
IL-6	Interleukin-6
IL-8	interleukin-8
IL-10	Interleukin-10
IL-17	Interleukin-17
IL-17A	Interleukin-17A
IL-18	Interleukin-18
iNOS	Inducible nitric oxide synthase
iTreg	Induced regulatory T cell
LC3-I	Microtubule-associated protein 1 light chain 3, form I
LC3-II	Microtubule-associated protein 1 light chain 3, form II
LPS	Lipopolysaccharide
M1	M1-like macrophage phenotype
M2	M2-like macrophage phenotype
MAPK	Mitogen-activated protein kinase
M-CSF	Macrophage colony-stimulating factor
MEK	Mitogen-activated protein kinase kinase
MHC	Major histocompatibility complex
MMP-2	matrix metalloproteinase-2
MMP-3	Matrix metalloproteinase-3
MMP-9	Matrix metalloproteinase-9
MS	Mass spectrometry
mTOR	Mechanistic target of rapamycin
mTORC1	Mechanistic target of rapamycin complex 1
NADPH	Reduced nicotinamide adenine dinucleotide phosphate
NET	Neutrophil extracellular trap
NFATc1	Nuclear factor of activated T cells 1
NF-κB	Nuclear factor kappa B
NK	Natural killer
NKG2D	Natural killer group 2 member D
NLRP3	NLR family pyrin domain containing 3
NOX2	NADPH oxidase 2
Nrf2	Nuclear factor erythroid 2-related factor 2
OPG	Osteoprotegerin
p22phox	22 kDa phagocyte oxidase subunit
p38	p38 mitogen-activated protein kinase
p47phox	47 kDa phagocyte oxidase subunit
p62	Sequestosome 1
p65	Nuclear factor kappa B p65 subunit
PI3K	Phosphoinositide 3-kinase
PTGS2	Prostaglandin-endoperoxide synthase 2
RA	Rheumatoid arthritis
RA-FLS	Rheumatoid arthritis fibroblast-like synoviocytes
RANKL	Receptor activator of nuclear factor kappa B ligand
RASGRF1	Ras protein-specific guanine nucleotide-releasing factor 1
RGD	arginine-glycine-aspartic acid
RORγt	Retinoic acid receptor-related orphan receptor gamma t
ROS	Reactive oxygen species
SPR	Surface plasmon resonance
STAT3	Signal transducer and activator of transcription 3
STAT4	Signal transducer and activator of transcription 4
STAT5	Signal transducer and activator of transcription 5
SYK	Spleen tyrosine kinase
T-bet	T-box transcription factor TBX21
TAK1	Transforming growth factor beta-activated kinase 1
Th1	T helper 1 cell
Th17	T helper 17 cell
TNF-α	Tumor necrosis factor alpha
TRAP	Tartrate-resistant acid phosphatase
Treg	Regulatory T cell
